# Reproduction of patterns in melanocytic proliferations by agent-based simulation and geometric modeling

**DOI:** 10.1371/journal.pcbi.1008660

**Published:** 2021-02-04

**Authors:** Günter Schneckenreither, Philipp Tschandl, Claire Rippinger, Christoph Sinz, Dominik Brunmeir, Nikolas Popper, Harald Kittler

**Affiliations:** 1 Institute of Information Systems Engineering, TU Wien, Vienna, Austria; 2 Institute of Analysis and Scientific Computing, TU Wien, Vienna, Austria; 3 dwh simulation service, dwh GmbH, Vienna, Austria; 4 Department of Dermatology, Medical University of Vienna, Vienna, Austria; H. Lee Moffitt Cancer Center and Research Institute, UNITED STATES

## Abstract

Spatio-temporal patterns of melanocytic proliferations observed in vivo are important for diagnosis but the mechanisms that produce them are poorly understood. Here we present an agent-based model for simulating the emergence of the main biologic patterns found in melanocytic proliferations. Our model portrays the extracellular matrix of the dermo-epidermal junction as a two-dimensional manifold and we simulate cellular migration in terms of geometric translations driven by adhesive, repulsive and random forces. Abstracted cellular functions and melanocyte-matrix interactions are modeled as stochastic events. For identification and validation we use visual renderings of simulated cell populations in a horizontal perspective that reproduce growth patterns observed in vivo by sequential dermatoscopy and corresponding vertical views that reproduce the arrangement of melanocytes observed in histopathologic sections. Our results show that a balanced interplay of proliferation and migration produces the typical reticular pattern of nevi, whereas the globular pattern involves additional cellular mechanisms. We further demonstrate that slight variations in the three basic cellular properties proliferation, migration, and adhesion are sufficient to produce a large variety of morphological appearances of nevi. We anticipate our model to be a starting point for the reproduction of more complex scenarios that will help to establish functional connections between abstracted microscopic behavior and macroscopic patterns in all types of melanocytic proliferations including melanoma.

## Author summary

Clinicians and pathologists use pigmentation patterns to classify melanocytic nevi into subgroups and to differentiate nevi from melanoma. The mechanisms that produce these patterns are poorly understood. Here, we reproduce the main patterns of nevi in computer simulations using an abstracted model of individual cell behavior. We developed a novel geometric approach for reconstructing the microanatomy of the epidermis in silico and to model the interaction of cell-agents with the physiological environment. We generated visual representations of simulated populations of melanocytes and demonstrate that the interplay of the collective behavior of individual cells and the physiologic microenvironment is responsible for the typical patterns of melanocytic lesions. Further, we show that the variations of nevi observed in vivo can be reproduced by simple agent-based models that depend on a few key parameters that reflect basic cellular functions such as proliferation, migration, and adhesion. Our simulations confirm existing assumptions about nest formation in melanocytic lesions and offer new insights and testable hypotheses for important biologic phenomena such as senescence and tumor progression. Furthermore, synthetic nevi created by our model may serve as training cases for machine learning algorithms.

## Introduction

Melanocytic nevi are common benign skin lesions composed of melanocytes. They can be congenital (visible at or shortly after birth) [1, 2] or acquired. Most acquired nevi occur in the basal layer of the epidermis (the dermo-epidermal junction), which is the physiologic microenvironment of melanocytes in the skin. Clinicians use a small handheld instrument, the dermatoscope, to examine nevi and divide them into subgroups according to their dermatoscopic patterns. The two main mesoscopic patterns of nevi are the reticular and the globular pattern [3]. From a histopathologic point of view, the reticular pattern is characterized by the spread of single melanocytes or small chords of melanocytes that are evenly distributed along the basal layer of the epidermis [4]. The globular pattern, on the other hand, is characterized by the formation of large epidermal nests of melanocytes [5, 6]. Traditionally, the behavior of melanocytes and the mechanisms of nest formation have been studied in vitro in two-dimensional cell cultures. Most research focused on melanoma, which result from the malignant transformation of melanocytes. In vitro 3D models of melanoma, such as melanoma spheroids embedded in extracellular matrix or organotypic skin reconstructs, have been developed to better mimic the three-dimensional microenvironment. In vivo melanoma models relied on mouse models such as tumor xenografts in immunocompromised mice [7], genetically engineered mice with conditional melanocyte-specific expression of the BRAF V600E oncogene [8], or mice with induced expression of the BRAF V600E oncogene by topical application of tamoxifen [9]. There is, however, still a need for more physiologically relevant models of the formation of nevi and melanoma in human skin.

Nevi and melanoma share common features including molecular changes, microscopic characteristics, and mesoscopic and macroscopic patterns [10]. Like most melanomas, most nevi are clonal proliferations of BRAF V600E/K mutant melanocytes [11]. Other activating mutations such as NRAS, GNAQ, GNA11, HRAS, and BAP1 mutations are less frequent than BRAF V600E/K, at least in acquired nevi. The type of mutation impacts the phenotype of melanocytes, and the mesoscopic and macroscopic patterns of nevi. Kiuru et al., for example, linked genotype with phenotype by demonstrating that epidermal melanocytes are more often arranged in nests in nevi with BRAF V600E mutations than in nevi that lack this mutation [12]. The formation of nests indicates progression of both types of melanocytic proliferations, of nevi and of melanoma [1, 2]. The presence and arrangements of nests is a key criterion for the diagnosis of melanocytic proliferations [13, 14] and larger nests are associated with faster growing nevi and more aggressive melanoma [5].

The molecular basis of cell-microenvironment interactions of melanocytes during nevogenesis including nest formation is incompletely understood. During nevogenesis melanocytes show complex self-organizing cooperative behavior. Our approach is to study it from a mesoscopic and macroscopic pattern driven point of view, and not from the molecular perspective. In theory, all differences between mesoscopic and macroscopic patterns of nevi should be attributable either to intrinsic factors that control basic functions of melanocytes such as proliferation [13], migration [14], cell-cell or cell-matrix interactions [15, 16], differentiation [17], and survival [18]; or to extrinsic factors such as the microanatomy of the skin [19–22]. Ultimately, to study hypotheses and basic functionalities about the connections between the meso- and macroscopic scale, it is our aim to map an abstracted and simplified version of this complex biological system in silico.

In general, computational simulation of cellular populations is considered a viable approach in many research areas of biology and medicine and a number of adaptable programming environments [23–27] have been developed in this regard. The proposed modeling techniques [26, 28] range from compartment models, over cellular automata [29, 30] and agent- or entity-based models [23, 27, 31, 32] to partial differential equations [28, 33]. Whereas compartment models and differential equations are suitable for describing aggregate quantities, entity-based models simulate cells on an individual basis. In the latter case the formalization of cellular processes and interactions can be more direct and versatile, but in turn, global or macroscopic patterns must emerge from microscopic dynamics. Computational models for simulating melanocytic proliferations on a micro- and mesoscopic scale have been described for histologic sections [34, 35], isolated cell colonies [30, 33] and local subpopulations in abstracted skin models [36, 37]. Computational simulation has also been used to show that the arrangement of mesoscopic structures may produce corresponding macroscopic pigmentation patterns. Using cellular automaton models derived from a continuous reaction-diffusion system, Manukyan et al. reconstructed the emergence of pigmentation patterns in lizard skin [38]. In a similar way, Volkening et al. and Owen et al. investigated pigment patterns in zebrafish by agent-based simulation of cellular interaction [39, 40]. Analogously, we hypothesize that nevus patterns emerge from nonlinear dynamical microscopic systems of cell-cell and cell-matrix interactions that can be simulated by abstracted mathematical models of basic cellular functions. We developed a mathematical model and simulation approach that connect abstracted microscopic dynamics of individual melanocytes in a realistic three-dimensional geometric model of the physiologic microenvironment with the morphologic appearance of nevi observed by dermatoscopy [41, 42] and histopathology [43].

**Fig 1:**
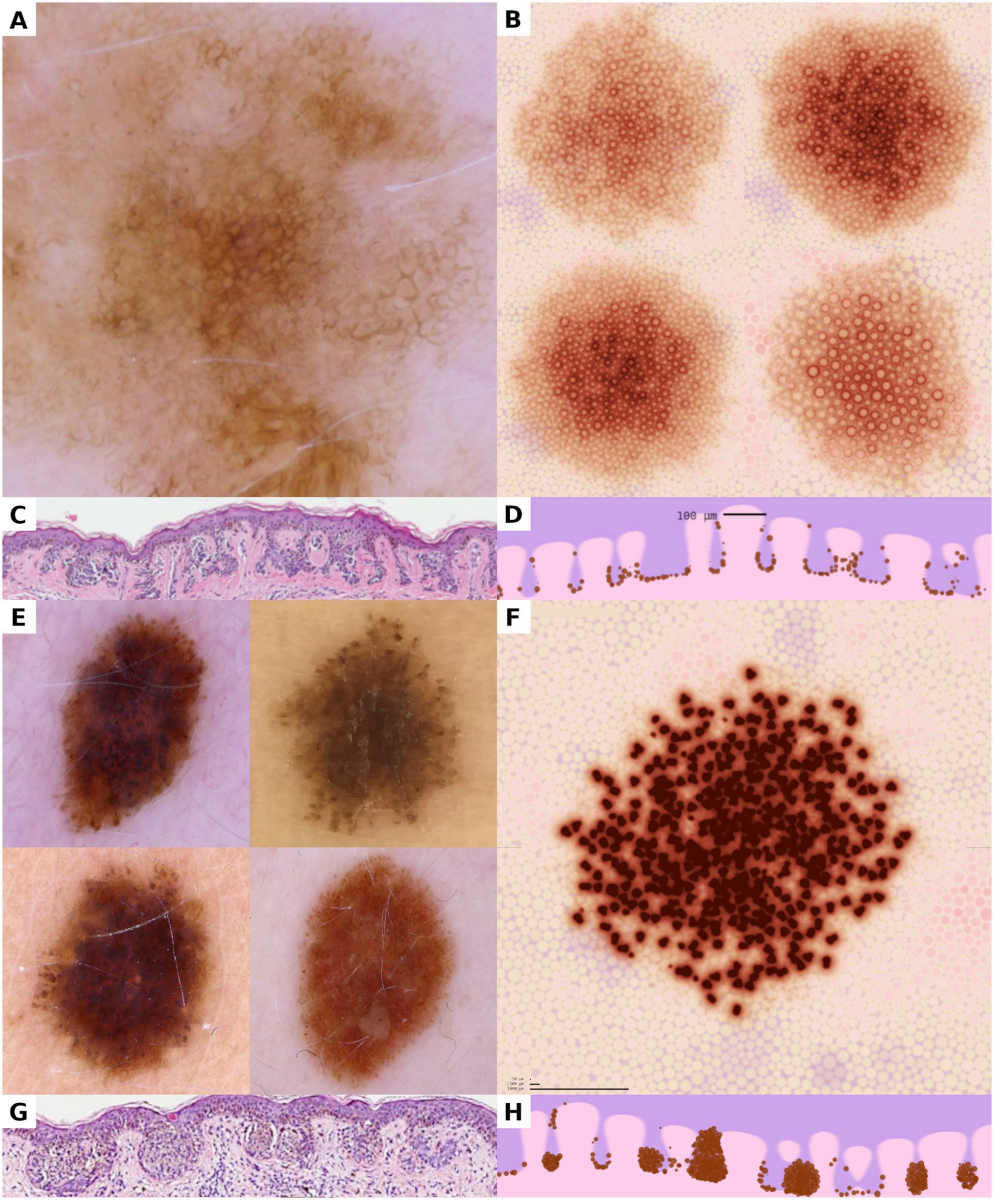
Juxtaposition of dermatoscopic and histopathologic images of real nevi and visualizations of simulated melanocyte populations. (A, B, C, D) A dermatoscopic photograph of a real nevus (A) and visual representations of corresponding simulated nevi (B) show that the reticular pattern can be reproduced in dynamic simulations. The preferential arrangement of melanocytes at the base of the rete ridges, as visible in histopathologic sections (C), can be reproduced in simulations and manifests in the visual representations (D). (E, F, G, H) Melanocytic nests appear as small brown globules in dermatoscopic images of globular nevi (E). The formation of nests in simulated populations yields the same globular pattern in visual representations (F). Vertical aspects of the dermo-epidermal junction with melanocytic nests is demonstrated in histopathologic sections of real nevi (G) and corresponding views obtained from our simulation (H). All horizontal images show a region of 5,000 μm × 5,000 μm; all vertical images represent 1,200 μm × 250 μm.

In the *Results* of this paper we present our motivation for the abstraction of the biologic system, introduce the layout of our conceptual and technical model and demonstrate how the two most common patterns, the reticular and the globular pattern, emerge in silico. In particular, we demonstrate how visualizations of simulation results in the style of dermatoscopic and histopathological images as shown in Fig 1 can be used to validate our model and how our simulations comply with the growth dynamics of real nevi. The *Discussion* contains a summary of the background, purpose and construction of our modeling and simulation approach. We discuss biological interpretations and explanations of certain phenomena of synthetic nevi and give an outlook on future applications and developments. In *Methods* we present a more detailed description of our mathematical models including parameterization and describe the visual rendering techniques. In the *Supporting Information* we provide the complete parameterization, additional model analysis, a variety of visual animations, and details of the implementation.

## Results

### Abstraction of the biological system

The microphysiological environment of the dermal-epidermal junction is the natural habitat of acquired nevi and the origin of most melanomas. The microanatomy of this area is governed by the characteristic shape of the basal membrane, a collagen type IV matrix interfacing both compartments [44, 45]. Normal melanocytes express CCN3, a matricellular protein that inhibits melanocyte proliferation and stimulates adhesion to collagen type IV. As a consequence, melanocytes are situated in the basal layer of the epidermis under physiologic conditions. In nevi, attachment of melanocytes to collagen IV is mediated through DDR1, which is under the control of CCN3 [46, 47]. Hence, we assume that the migration of nevus forming melanocytes in the basal layer can be described by mechanisms that let cell-agents *glide* along the membrane surface. Modeling the basement membrane in a differential geometric approach allows us to simplify the movement of nevus forming melanocytes as abstracted velocity vectors, while, at the same time, enables smooth and continuous migration along the complex geometric shape of the membrane.

Under physiologic conditions there exists a strong symbiosis between keratinocytes and melanocytes [16, 48, 49], which plays a crucial role in the proliferation and migration of melanocytes in the basal layer. In view of the feasibility and tractability of the individual-cell simulation approach, we integrate relevant interaction with keratinocytes in the abstracted behavior of melanocyte-agents. Thus, we simulate only nevus forming melanocytes but not keratinocytes or the background of normal melanocytes. Similarly, in our approach melanocyte-melanocyte interaction is not modeled on a biomolecular level [16] but abstracted in the movement dynamics of cell-agents (attraction, repulsion, collision). Hence, forces and collisions among cells in our model must not be understood as physical quantities (conservation of momentum, etc.), but as abstracted mechanisms that reproduce a larger set of complex processes [14, 15]. Detailed discussion of different configurations of cell motility are found in the following sections on the formation of the reticular and globular pattern.

Because our simulation approach is characterized by heavy abstraction of microscopic and cellular processes, also the parameters must be simplified and aggregate representations of biological quantities. For instance, we model the division of cells as discrete stochastic events that are parameterized with probabilistic likelihoods [30, 36]. In reality, cell division is a complex biological process that is not instantaneous but controlled by a large number of molecular reactions. To map the most crucial (extracellular) influences on the proliferation rate, we use heuristic damping factors that reproduce the biological effects we know or expect to exist in the biological system. This is, for instance, a reduction of the proliferation rate in areas with high cell density via contact inhibition [50] or induction of senescence of cells depending on the generation number (number of past divisions) due to telomere shorting [51].

### Geometric model of the epidermal microanatomy

The spatial domain of simulated cell-agents is a cuboid section of virtual tissue that is separated into a dermal and epidermal compartment by a vaulted surface representing the basement membrane. We developed a detailed three-dimensional geometric model of the basement membrane to reproduce the typical microanatomy. For simplicity, skin appendages (hair follicles and eccrine glands) are not included. Dermal papillae are modeled as circular evaginations and their lateral S-shape is obtained from the revolution of parameterized *shape curves*. We formalize the basement membrane as a two-dimensional composite differentiable manifold.

**Fig 2:**
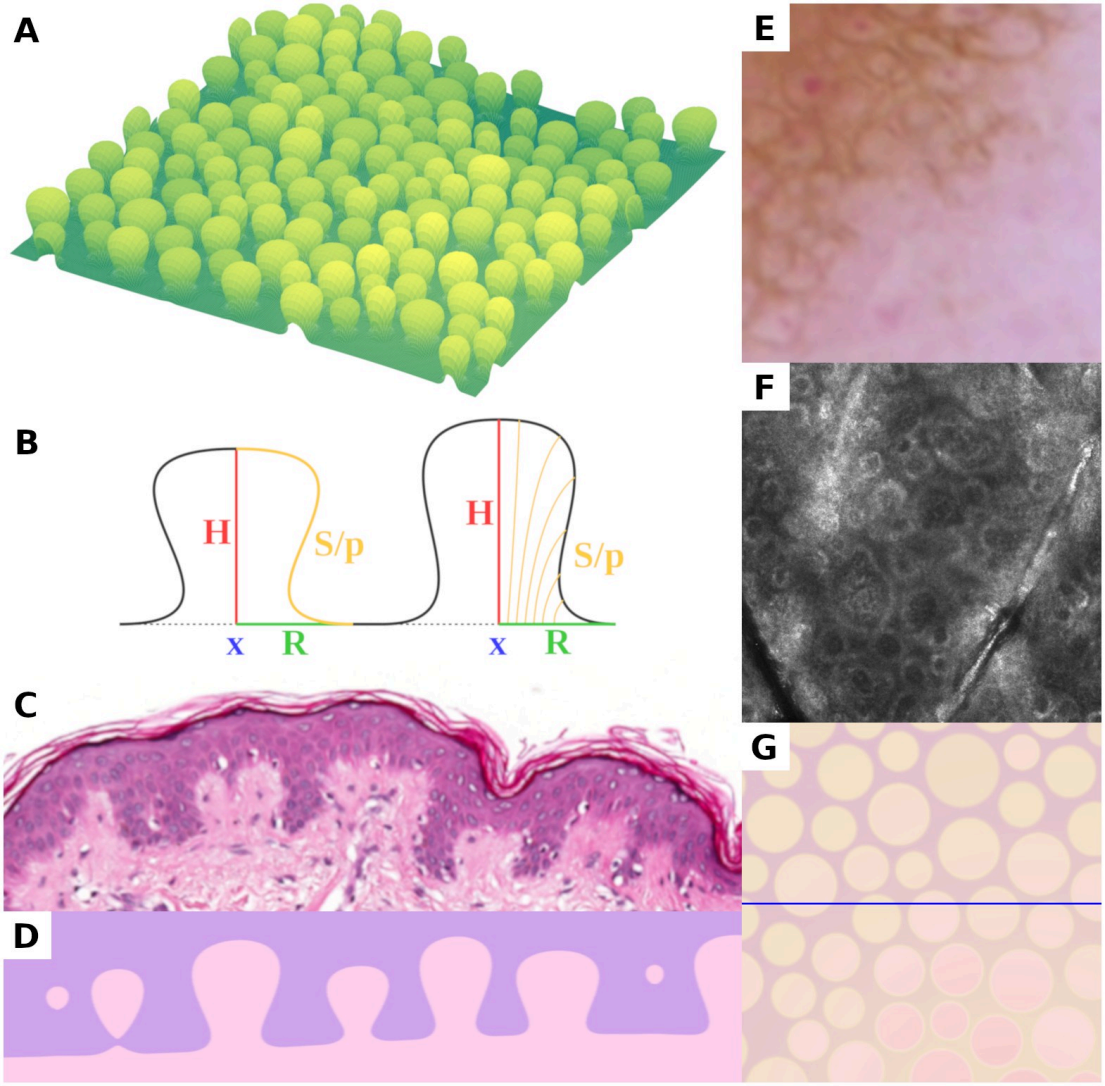
Presentation of the three-dimensional geometric model of dermal papillae and the dermal-epidermal junction. (A) Rendering of a computer generated basement membrane section of 1,000 μm × 1,000 μm. (B) Schematic lateral section of the geometric papilla model (location *x*, height *H*, radius *R*, scalar shape parameter *p* and derived shape curve *S*). (C, D) Routine hematoxylin and eosin stained histopathologic section of epidermis with dermo-epidermal junction measuring roughly 600 μm in width (C) and corresponding visual representation of computer generated tissue (D). (E, F, G) Horizontal views of the epidermal microenvironment as seen with dermatoscopy (E), in vivo confocal microscopy (F), and visualization of the geometric model (G), each measuring 500 μm × 500 μm. The lighter circular areas in (E) and (G) correspond to dermal papillae, which appear as hyporeflective areas surrounded by a rim of hyperreflective cells in confocal microscopy images (F). All three horizontal views show a square section with 500 μm side length. The horizontal line in (G) indicates the sectional plane in (D).

In order to reproduce realistic tissue anatomy, we inferred statistical distributions of shape parameters and papilla density from measurements of histopathologic sections, and from in vivo images obtained by dermatoscopy and laser scanning confocal microscopy. Due to the absence of skin appendages, the number of dermal papillae per area is slightly greater in our model than in vivo. Histopathologic sections of excised tissue are distorted by shrinking [52], which has been taken into account in the parameterization of the geometric model (Fig 2).

### Agent-based model for migration and proliferation

We conceptualize melanocytes as three-dimensional spherical objects with internal states such as size and position that are subject to abstracted attractive, repulsive, and random movement forces. In concordance with the natural behavior and localization of melanocytes, the geometric model of the basement membrane is used to constrain the movement of simulated cells to the basal layer of the epidermis, and to prevent emigration from the epidermal compartment. Cellular processes such as division and differentiation are modeled as stochastic events. The temporal evolution of the simulated melanocyte population is obtained from an iterative algorithmic scheme.

**Fig 3:**
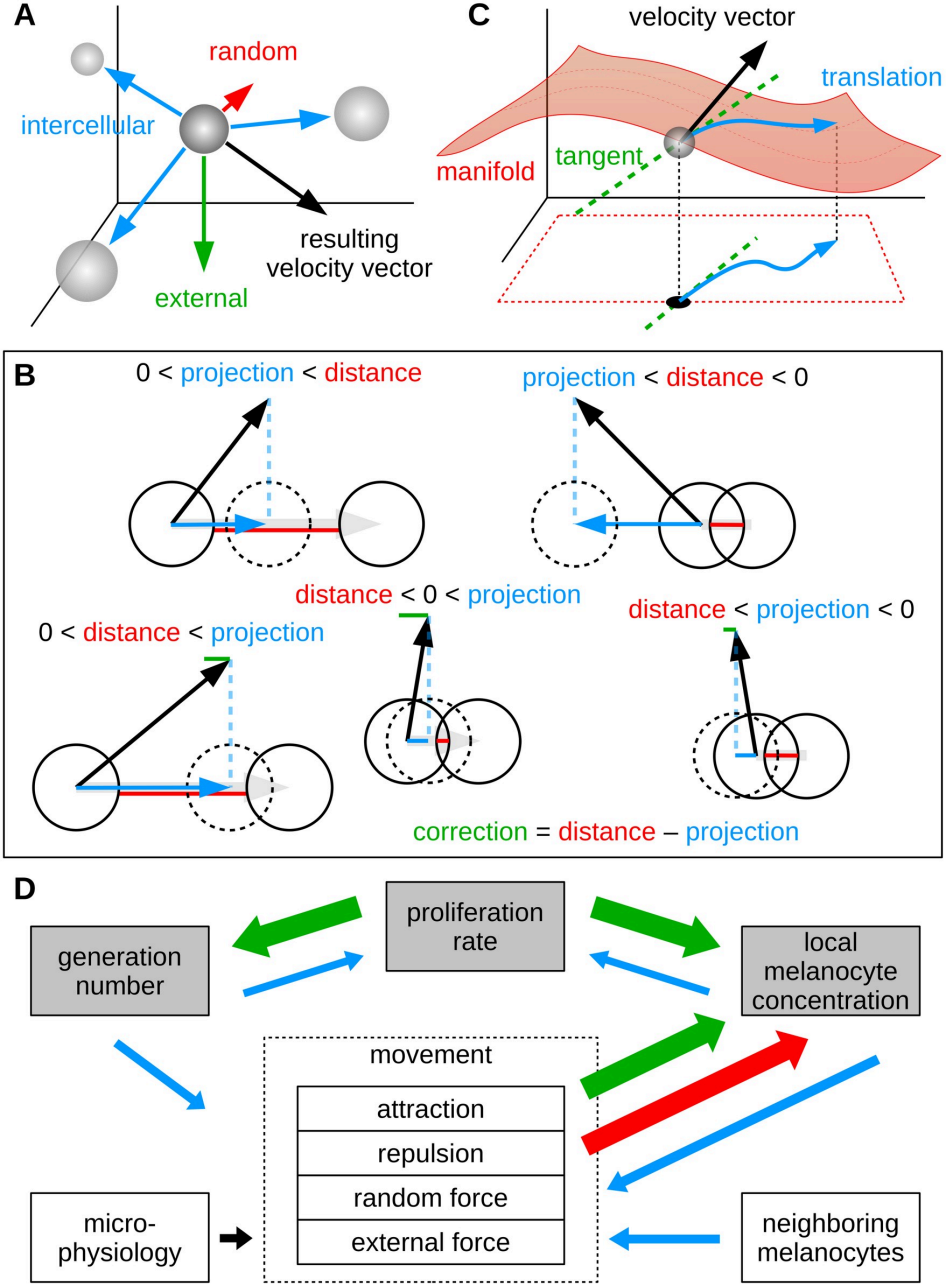
Schematic outline of the agent-based model. (A) Abstracted intercellular forces (attraction and repulsion), random forces and external forces impose a velocity vector on each individual cell. (B) To minimize the overlap of spherical cells, a simplistic corrector algorithm is applied on the resulting velocity vector (for clarity shown in two dimensions). In all cases where the anticipated new position of a cell results in overlap with a neighboring cell, a certain (projected) component of the velocity, which we call the correction, is subtracted from the vector. Due to iterative application, this procedure ensures that the total overlap of simulated cells is minimized. (C) The corrected three dimensional velocity vector is projected into the coordinate space of the manifold model. By solving the geodesic differential equation, motion along the membrane surface can be simulated. (D) Outline of dynamic effects in the agent-based model. Large arrows (green and red) indicate effects that emerge naturally from the structural concept and the resulting internal logic of the model. Increase of the generation number of cells and of the local density result from cell division. Whereas attractive forces among melanocytes lead to higher density values, repulsion and random motion (diffusion) decrease the local concentration. Effects that do not emerge automatically, are controlled by functional influences (small arrows, blue).

To prevent two cells from occupying the same space, we use a technical mechanism that relaxes the force vectors of colliding spherical agents. Interaction with the manifold (basement membrane) is simulated by geometric transformations of velocity vectors and geodesic displacements. Fig 3A–3C presents a schematic of the movement model as a combination of movement forces, collision handling and geodesic translations. We introduce relatively unconstrained movement at a later point to simulate the scenario of nest formation. To modulate the movement forces and the likelihoods for cell division and differentiation on an individual basis, we use a measure of the local concentration of melanocytes and the cell generation number. Hence, the parameterization of the dynamic agent model consists of various stochastic quantities and control functions of the form

$$\begin{array}{cc} \text{generation}\times \text{density} & ⟶\text{multiplicative}\;\text{damping}\;\text{factor}\\ \text{distance}\times \text{generation}\times \text{density} & ⟶\text{force}\;\text{vector} \end{array}$$

where the latter is to be understood pairwise (cells and their neighbors). In Fig 3D we illustrate the conceptual layout of this approach and differentiate between dynamic effects that emerge naturally as a result of the model structure and additional effects that we introduced based on further abstracted biological assumptions. The mathematical realization of our simulation model and the derivation of individual model parameters is presented in *Methods*.

### Emergence of the reticular pattern

**Fig 4:**
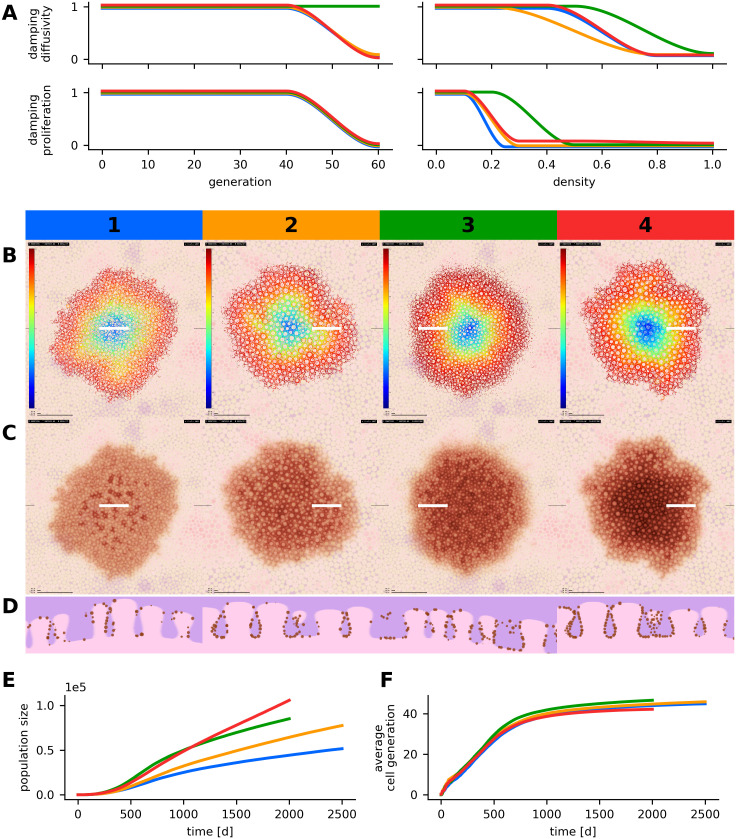
Parameter variation in simulated reticular nevi. The results of four simulations runs with different parameter configurations are compared. Each scenario corresponds to a number (1-4) and a specific color (blue, orange, green, red). (A) Configuration of generation and density dependent damping factors. For each of the four scenarios, four parameter curves are displayed in the respective colors. The parameter curves describe the damping factors for proliferation and diffusivity depending on generation number and local density. (B, C, D) Visualization of the spatial cell generation profiles (B)—the color map refers to generation numbers between 0 and 60—, simulated dermatoscopic views (C) and histologic views (D). All images depict the state of the simulated lesions after 2,000 or 2,500 days, horizontal images display a region of 5,000 μm × 5,000 μm and white markers indicate the location of the histologic section. (E) Temporal evolution of the population size. (F) Temporal evolution of the average generation number in simulated melanocytes. Scenarios 2 and 4 use a tissue model with larger dermal papillae. An additional downwards force was applied in scenarios 2, 3 and 4. Different instances of growth arrest and corresponding radial generation profiles can be compared in scenario 1 and 3. The parameterizations of all four scenarios are listed in Table B in S2 Text.

We show in a first experiment that our model can reproduce the connection of meso- and macroscopic traits in nevi with a reticular pattern. It is known that in vivo the reticular pattern reflects the microanatomy of the dermal-epidermal junction. A relative higher density of melanocytes and pigment on the lateral surfaces of dermal papillae produce ring shapes in horizontal dermatoscopic and microscopic images [6]. In our model we were able to reproduce this configuration with a small set of rules, which state that migration of cells is driven by random forces only. A selection of model parameterizations that produce reticular nevi with different features such as central hyperpigmentation, hypo-hyperpigmentation and homogeneous pigmentation as described by Hofmann-Wellenhof [53] are presented in Table B in S2 Text. Plots of the according functional damping factors as well as visual and quantitative simulation results are presented in Fig 4. A video showing the temporal evolution of a simulated reticular nevus is provided in S2 Video.

We conclude that the reticular pattern results from a complex interplay of simple cellular dynamics (proliferation and diffusive migration) and the microanatomy of the dermo-epidermal junction (cell-matrix adhesion). A dynamic balance between proliferation and migration preserves a density equilibrium during growth. In low density areas, undamped proliferation increases the number of melanocytes, whereas in medium density areas, diffusive migration leads to the emigration of cell-agents. In high density areas, the packing of cell-agents impedes their displacement. Technically, the collision mechanism cancels out a large amount of the movement forces in individual cell-agents. The resulting *freezing effect* corresponds to increased melanocyte-melanocyte bonding [16]. However, to prevent unrealistic jittering of simulated cell-agents and to account for the presence of other cells that are not displayed by our model (e.g. keratinocytes), we explicitly dampen the initial movement forces in high density areas. In high density areas we also reduce the proliferation rate of melanocytes to mimic the biologic effect of contact inhibition [50, 54]. We further configure our model in such a way that proliferation and migration degrade with higher generation numbers, which corresponds to oncogene-induced senescence in vivo [55]. As a consequence, only a limited *proliferative potential* [29, 32] is available and the simulated nevus reaches a steady state and a final size as it is observed in real nevi. A certain *proliferative potential* in the confined center region cannot be exhausted because higher density prevents the proliferation of cells (compare the concentric density and generation profiles in Fig 4). Accordingly, the generation number is highest in the periphery of the nevus. In S3 Text we investigate mathematical models for the growth of simulated populations in abstracted non-spatial scenarios.

Different shapes and borders of simulated nevi, which mirror the variations seen in real nevi, are a consequence of stochastic events during simulation. However, the morphological appearance and quantitative characterization of simulated nevi is stable with respect to parameterization (S5 Text). That is, repeated simulation runs with the same parameterization produce adequately similar results.

In histologic sections of reticular nevi, melanocytes are preferentially situated at the base of the rete ridges and avoid the tips of dermal papillae. The reasons for this phenomenon are not completely resolved [56]. In our computer simulations, this pattern is automatically reproduced to a certain extent with above model configuration. This may indicate that this phenomenon emerges as a consequence of the microanatomy of the epidermis. A pronounced expression of this behavior in simulated melanocytes can be obtained by imposing a slight downward force on the cells. In this case, a higher density of adhesion molecules may be responsible for the preferential attachment of nevus forming melanocytes in this part of the epidermis. In S1 Text and S1 Video we investigate how the movement behavior of melanocytes and the particular geometric form of the basement membrane jointly produce this vertical inhomogeneity.

### Nest formation and emergence of the globular pattern

The biological and molecular mechanisms that lead to the formation of nests are not fully resolved. However, it is known that the activation of certain adhesion molecules plays a pivotal role [15, 30, 31, 36]. Here, we interpret the emergence of globular nevi as the result of the occurrence of a nest-forming phenotype. In silico, commencing from the basic reticular configuration we introduce a certain cellular differentiation to simulate melanocytic nests as local strains of highly proliferative and slightly adhesive cells. This approach aligns with results from computational simulations that attribute the formation of melanocytic nests to proliferative activity rather than migration [30, 36].

Technically, differentiation into a globular strain occurs randomly during the division of melanocytes. The membership to a globular strain is inherited during subsequent cell divisions such that each nest stems from a single cell [29]. All members of a nest are allowed to detach from the basement membrane and the geodesic movement model (restriction to the basal layer) is replaced by unconstrained movement. As a consequence, adhesion among melanocytes in the same strain, which is simulated by local attractive forces, leads to spherical nests. Slight repulsive forces among cells, which are not in the same strain, prevent the *fusion* of different globules. External forces are used to prevent globules from entering the dermal compartment and also simulate cell-matrix adhesion in such a way that nests are predominantly located in the lower regions of the rete ridges [57]. Hence, in silico, the formation of nests is associated with a distinct differentiation and additional migratory dynamics whereas the behavior of single cells is analogous to the reticular scenario (compare the parameter configurations in Table B and C in S2 Text).

The size and temporal evolution of melanocytic nests was investigated in experiments in vitro and in silico [30, 36]. In our model the size and temporal evolution of nests is stochastically controlled through proliferation and emigration (cells leaving the nest). The likelihood of these events depends on the within-strain generation number of individual cells (number of divisions since the initial differentiation). Emigrated cells return to the behavior of the default population. If all cells of a globular strain emigrate, the nest is dissolved. In S4 Text and S3 Video according mathematical models for the size of simulated melanocytic nests are formalized and reproduced in simulations.

**Fig 5:**
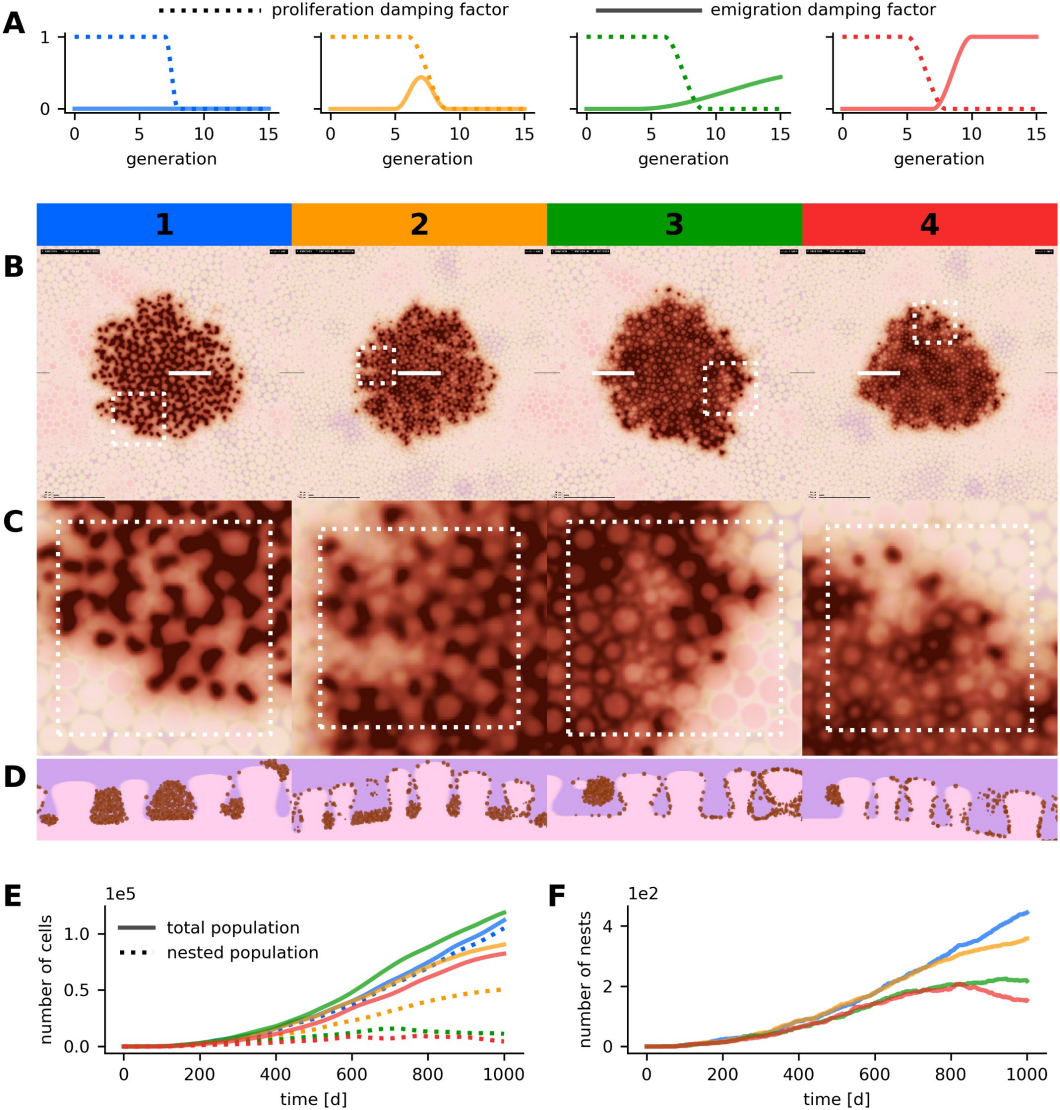
Parameter variation in simulated nevi with the globular pattern. Results of four simulation runs with different parameter configurations are compared. Numbering and color coding is analogous to Fig 4. (A) Configuration of the emigration and proliferation factors for each parameter scenario. (B, C, D) Dermatoscopic visualization of the cell population (B) with enlarged region (C) and histologic views (D). All images depict the state of the simulated lesions after 1,000 days, horizontal images display a region of 5,000 μm × 5,000 μm and white markers indicate the position of the enlarged region and of the histologic section. (E) Temporal evolution of the total and nested population size. (F) Temporal evolution of the number of globules. Simulated dermal tissue with larger papillae was used in scenarios 1 and 3. A detailed overview on the parameterization is available in Table C in S2 Text.

In Table C in S2 Text we present four slightly different parameterizations of this extended model. The parameterizations correspond to the four simulation scenarios in Fig 5 and demonstrate that a broad range of synthetic globular nevi can be generated by slight variation of a limited set of parameters. Furthermore, the formation, size and distribution of globules is similar to real nevi and repeated simulations with the same parameterization lead to quantitatively and visually similar results (S5 Text).

If proliferation within nests is high (i.e. large within-strain generation numbers allowed) and emigration is low, globules are larger and the density of melanocytes in the nests is higher. An increased emigration factor allows melanocytes to steadily detach from nests and results in smaller globules and a larger population of single cells. The dermatoscopic and the histopathologic representations of scenarios 1 and 2 in Fig 5 reflect the expected behavior. If emigration does not decline with increasing generation number or exceeds a critical threshold with respect to proliferation, globules dissolve over time. In scenarios 3 and 4 in Fig 5 this behavior was reproduced. In all four scenarios, the nests reach their maximal size after approximately 100 days. Average nest sizes range between 200 and 500 cells.

**Fig 6:**
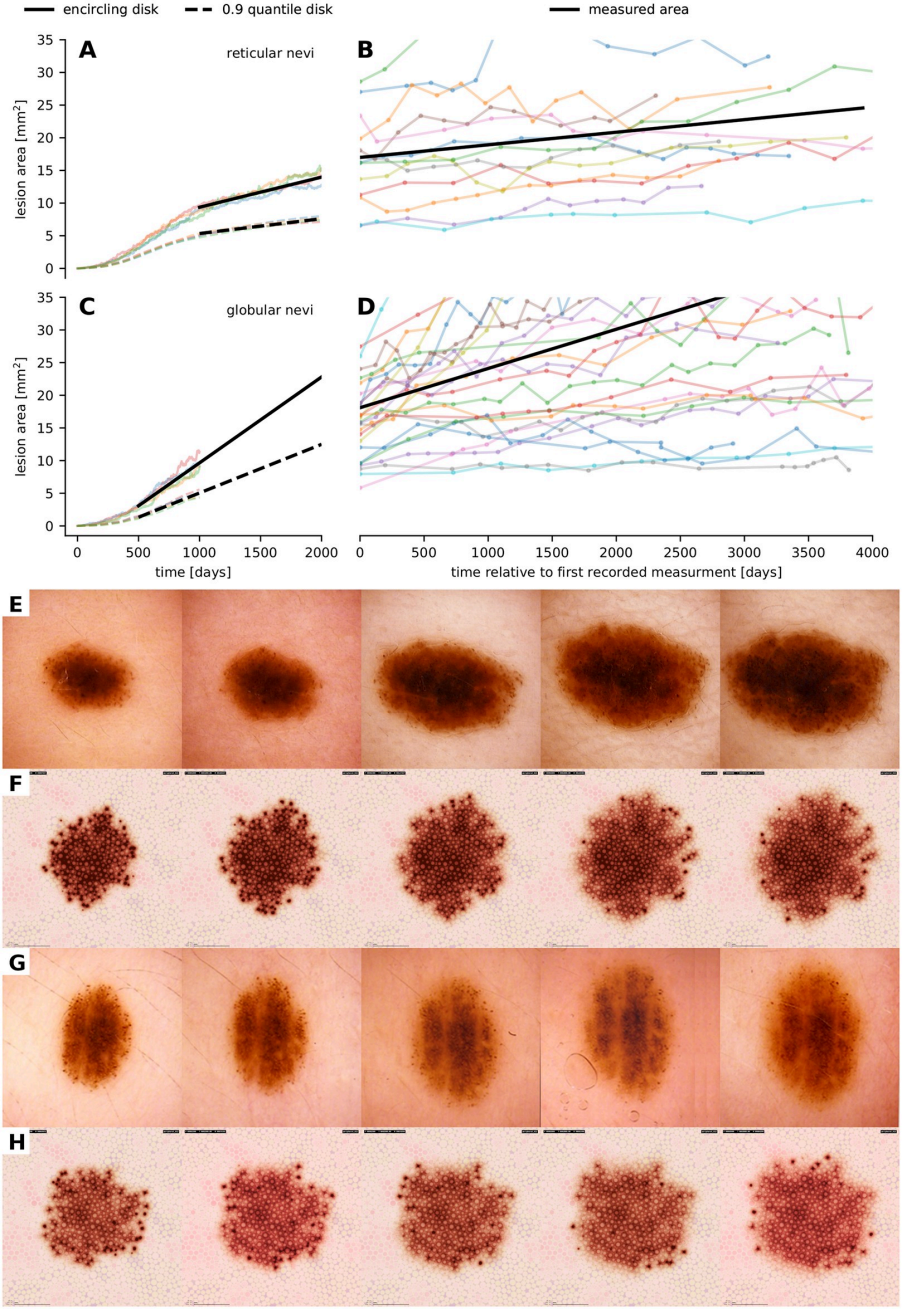
Comparison of the growth dynamics of nevi as documented in clinical imagery and generated by our model. (A, B, C, D) Comparison of the spatio-temporal evolution of the areal size of four simulated reticular (A) and globular (C) nevi as well as 13 reticular (B) nevi and 25 globular (D) nevi that were retrieved retrospectively from collected anonymized sets of digital dermatoscopic images. We used linear interpolation to quantify the growth dynamics in silico and in vivo. Black lines show the averaged linear growth dynamics. (E, F, G, H) Temporal evolution of the dermatoscopic exposition of real (E, G) and simulated (F, H) nevi with a peripheral rim of globules. Dissolving melanocytic nests in the center region yield a reticular pattern as seen in all four image series. In real nevi, additional to biological variations in the mesoscopic behavior of cells, tension forces such as relaxed-skin-tension, Blaschko and Langer lines can be the cause for morphological eccentricity and border morphology.

In our simulations not only the size of nests but also the spatial distribution results from a balance between proliferation and emigration. If nests dissolve over time, the densely populated central part of the lesion, which has a low proliferative activity, is covered by a reticular pattern. The periphery, on the other hand, which has a relatively high proliferative activity, is typified by a globular pattern. This can be recognized in scenarios 3 and 4 in Fig 5 and corresponds to the active border of real growing nevi with the typical rim of globules in the periphery [5, 58] (see also Figs 1 and 6). As in real nevi, the peripheral globules indicate that the lesion is still growing.

### Temporal evolution and growth patterns

To further assess the plausibility of our simulation results, we compare the temporal evolution of simulated nevi with longitudinal clinical data. In particular, as a quantitative indicator, we correlate the evolution of the area occupied by simulated melanocyte populations with sequential area measurements of real nevi in retrospectively collected digital images of 25 globular nevi and 13 reticular nevi with more than 10 observations over time and an initial size of 30 mm^2^ or less [5, 58–60]. The area of lesions in dermatoscopic images was obtained by image segmentation with a pretrained fully convolutional network where ResNet34 layers are reused as encoding layers of a U-Net style architecture [61] and subsequent conversion of pixels into square millimeters according to the resolution of the used videodermatoscope. The area of artificial lesions was measured from the positioning of all simulated melanocytes by calculating the area of the smallest enclosing disk and of the 0.9 quantile error disk. Due to the limited visible pigmentation effect of sparse outlying melanocytes, the 0.9 quantile error disk is a better approximation of the areal size as detected in dermatoscopic images. In Fig 6A–6D we demonstrate that the simulation of the growth dynamics of reticular and globular nevi is in accordance with biology. We further quantified the slopes of the growth curves by linear least-square interpolation. The obtained slopes for simulated and real reticular nevi were 2.271 × 10^−3^ mm^2^ d^−1^ (standard deviation 0.364 × 10^−3^) and 1.928 × 10^−3^ mm^2^ d^−1^ (standard deviation 1.889 × 10^−3^) and for simulated and real globular nevi 7.442 × 10^−3^ mm^2^ d^−1^ (standard deviation 0.876 × 10^−3^) and 6.005 × 10^−3^ mm^2^ d^−1^ (standard deviation 6.099 × 10^−3^). A unpaired t-test shows that globular nevi grow faster than reticular nevi in simulation (P = 0.398 × 10^−3^) and in vivo (P = 4.365 × 10^−3^). Futhermore, we see that for both patterns there is no significant difference between the linear growth approximations of real and simulated nevi (P = 0.546 in the reticular scenario and P = 0.277 in the globular scenario). The growth data is available in S2 Table.

In Fig 6E–6H we further qualitatively compare the mesoscopic and macroscopic dynamics of real nevi and simulated nevi composed of melanocytes that are able to form nests. When parameters are set in such a way that the probability of emigration (cells leaving the nest) is high, a rim of globules forms in the periphery and the reticular pattern is maintained in the center. This pattern is typical for growing nevi and can be reproduced in simulated lesions. If the probability for emigration is low, nests are maintained in the center. Videos showing the temporal evolution of a simulated globular nevus and a simulated nevus with peripheral globules are available in S4 and S5 Videos.

## Discussion

Many molecular processes in the formation and evolution of melanocytic lesions are incompletely understood. In particular, a broad range of the cellular dynamics observed on the mesoscopic scale (migration, proliferation, binding) lack full biological explanation and insight. However, the connection between molecular processes, mesoscopic characteristics and resulting macroscopic patterns is a crucial aspect in clinical diagnosis. In this paper, we present a conceptual and mathematical model that simulates the proliferation and migration of melanocytes in the dermal-epidermal junction and can reproduce macroscopic patterns as observed in dermatoscopy and histopathology. The aim of this model is to provide and test hypotheses about the connections between the cellular dynamics of melanocytes and emerging meso- and macroscopic structures. It is our goal to make statements like “nests are more likely the result of proliferation than aggregation” or “diffusive movement behavior is sufficient to reproduce the spatio-temporal expansion of real nevi with the reticular pattern”. By this means we hope to gain new insight into the functional relations and the formation of patterns in nevi and melanoma.

Simulation models can only map a certain fraction or aspect of a complex natural system and it is necessary to define reasonable model boundaries in accordance with the intended scope of the model. Our approach is characterized by a parsimonious and highly abstracted description of the cellular dynamics [23, 29, 30, 33, 34, 36, 37] of melanocytes and the novel integration of a geometric replica of the microanatomy (see Results). Under cellular dynamics we understand abstracted movement and proliferation characteristics that equip melanocytes with a certain phenotype (*agent*). As a consequence, the behavioral spectrum of simulated cells can be defined by few mathematical formulas and numerical parameters that aggregate multiple biological quantities and processes (see Methods). It is however not our goal to predict the evolution of individual nevi or to reproduce the behavior of nevus forming melanocytes by simulating molecular processes.

To align and compare our simulation results with data obtained with the standard visual diagnosis methods, we developed visual rendering techniques that display simulated melanocyte populations in dermatoscopic and histopathologic image styles. We used this visual approach to fine-tune the parameters of our simulation model in order to reproduce the quantitative characteristics of emerging macroscopic patterns and the temporal evolution of real nevi [62]. Beyond the two main patterns, reticular and globular, the morphologic appearance of acquired nevi may vary with regard to pigmentation, shape, size, and border abruptness [53]. Partially in correlation with the emerging features, also the dynamic spatio-temporal expansion (growth) of melanocytic lesions can vary greatly. However, derived parameter values and ranges are plausible and in line with biological quantities encountered in the literature (see Methods). To further assert the plausibility and validity of our highly interconnected artificial system, we separated individual model components and analyzed them in conditioned environments. For instance, in S1 Text we discuss the impact of the particular geometric shape of the basal membrane in connection with different movement models (e.g. constrained diffusive motion) on the vertical distribution of simulated cells. We also compare the evolution of the population size in our model with unconstrained and non-spatial scenarios (S3 Text) and analyze the growth dynamics of individual melanocytic nests in simplified mathematical models (S4 Text). To ensure that simulation results are not tainted by artifacts in our models and algorithms and that biological parameters are reflected correctly, we tested for invariance with respect to different time increments (S6 Video). We performed repeated simulations with the same parameterizations and confirmed that the stochastic variations in our quantitative and qualitative simulation results are confined to a reasonable range (S5 Text).

In the most fundamental simulation scenario, we configure the behavior of cell-agents in order to reproduce—in combination with the geometric model of the dermal-epidermal junction—the macroscopic patterns of reticular nevi. We reconstruct the key assumption that the interplay of basic cellular dynamics with the microphysiological constraint (basement membrane) is the crucial driver for the emergence of this pattern and complex models of cellular behavior are not required (simplistic reproduction and migration behavior). Our data suggest that the reticular pattern emerges solely from proliferative melanocytes. In histopathologic sections of reticular nevi a higher number of melanocytes are present at the lower layers of the epidermis (the base of the rete ridges). We reproduce this arrangement of melanocytes to some extent by our basic model configuration as a result of the particular geometric shape of dermal papillae. However, to emphasize this inhomogeneous vertical arrangement of melanocytes, additional movement behavior is required, which indicates that melanocytes *actively* prefer this region. One explanation for this behavior could be that the density of adhesion molecules, which bind melanocytes to the basement membrane, is higher at the base of the rete ridges. We further observe that the generation number of simulated melanocytes is distributed in a concentric fashion with higher values in the periphery. This is in line with the frequent observation that visible malignant changes often start in the periphery of melanocytic lesions and with the model of stepwise tumor progression. Somatic mutations, such as those induced by UV-exposure, accumulate over time [29]. The mutational burden, which is generally high in melanoma, increases with each generation and peripheral melanocytes with a higher mutational burden are more likely to develop into malignant cells. In our model, reproductive activity of melanocyte-agents decreases due to two different mechanisms. A high generation number damps proliferation in the periphery of the simulated lesion. This mirrors growth arrest by telomere shortening in in vivo [51]. In the central region of simulated nevi, senescence is a result of high melanocyte density. This observation suggests that senescence requires intact cell-cell interaction between melanocytes [50].

Various simulation studies showed that in our model the emergence of the globular pattern requires additional cellular dynamics aside from division and attractive or repulsive forces. In particular, we came to the conclusion that—at least on a technical or phenomenological level—a certain differentiation has to occur. In line with existing studies [36], we simulate the formation of nests by a rapidly proliferating subpopulation of melanocytes, which is characterized by increased and exclusively mutual attractive forces (adhesion). The dissolution of globules is simulated by increased emigration of nested cells, which results in a mixture of the reticular pattern with a peripheral rim of globules during the growth phase of a nevus. Accordingly, the emergence, size and temporal evolution of nests is determined by a balance between within-nest proliferation and emigration. In general, large nests indicate an overall increased rate of proliferation, which is in line with the observation that real nevi and melanoma with large globules grow relatively fast [5, 58]. We further compared the temporal evolution of qualitative and quantitative features of simulated nevi with data obtained from sequential dermatoscopic examination. Our results show that slightly different mesoscopic behavior adequately reproduces the qualitative and quantitative differences between different phenotypic and morphological variants of nevi.

We demonstrate how an abstracted agent-based model of cellular dynamics in combination with a geometric model of the physiological microenvironment can reproduce biological processes and pattern formation on multiple scales of magnitude and time. The *emergent behavior* produced by our model mirrors complex biological processes. This simulation approach could provide a methodology to construct and systematically test new hypotheses about the formation and dynamics of melanocytic proliferations. For instance, the early phases in the evolution of skin lesions are rarely documented in clinical examination. Our approach could be of particular use to investigate this initial period by calibrating the model with intermediate to late phase sequential imagery and data [62]. Hypotheses constructed from our model could further serve as a starting point for future biomolecular studies. Vice versa, the inclusion of new and additional biomolecular findings could be used to improve the simulation of cell-cell interaction (e.g. bonding) in the future such that the underlying biological processes are reflected more explicitly. The model can also be extended by introducing particular germline and somatic mutations [13] that manifest in altered proliferative and migratory behavior (e.g. emigration from the epidermal compartment). Likewise, the reconstruction of the radial pattern in melanocytic lesions and the evolution of melanoma is left to future iterations of this model. By covering an even broader range of cellular dynamics and macroscopic patterns that occur in real nevi, a computational framework for the simulation of more general melanocytic lesions can be built.

Another possible future application of our model is to generate synthetic nevi that may serve as training cases for machine learning algorithms. Convolutional neural networks are increasingly used to support humans in the diagnosis of benign and malignant skin lesions in clinical practice [63]. One of the biggest issues facing the use of machine learning in image based diagnostic medicine is the lack of large, well annotated datasets. Currently, generative adversarial networks (GANs) are used to generate synthetic examples with the appearance of real images [64]. GANs are also used for augmentation of training set images, for example, to generate alternative versions of a real image. The disadvantage of synthetic images created by GANs is that they need real images as a starting point and that they cannot create new examples ex ovo. Synthetic nevi created by our model, on the other hand, are derived from simulated cellular behavior, and, like in real nevi, their variable appearance evolves from stochastic processes in combination with basic cellular properties such as proliferation, migration and adhesion. Combining our approach with GANs could open new exciting possibilities regarding the creation of synthetic images for machine learning purposes.

## Methods and models

In the following we present the complete mathematical formalization and parameterization of our conceptual model. In alignment with the general practice in the development of simulation models, we separate the implementation from the mathematical formulation. Implementation details can be found in S6 Text.

### Differential geometric model of the basal membrane

A two-dimensional manifold is a topological space, locally homeomorphic to the Euclidean space $R^{2}$. That is, there exists a set of continuous charts with continuous inverse, that map surroundings (e.g. disks) in the manifold *M* onto surroundings in $R^{2}$. More specifically, there exists a covering collection of open sets {*U*_*i*_}_*i*_ such that ⋃_*i*_*U*_*i*_ = *M* and corresponding local charts *φ*_*i*_: *U*_*i*_ → *V*_*i*_ where *V*_*i*_ are open sets in $R^{2}$. The collection {(*U*_*i*_, *φ*_*i*_)}_*i*_ is called an atlas. A manifold is *k*-times differentiable, if all chart-compositions $\varphi_{j}^{-1}\circ \varphi_{i}{|}_{U_{i}\cap U_{j}}$ are *k*-times continuously differentiable. This *compatibility property* of the atlas is required for defining tangent spaces and vector fields on *M*. For a complete introduction to manifold theory, we refer to [65–67].

We start with a subset *M*_0_ of the hyperplane $\left\{\left(x,y,z\right)|z=0\right\}\subset R^{3}$ representing a planar square section of the basal membrane without protruding dermal papillae. In combination with the projection mapping $\pi :R^{3}⊃M_{0}\to R^{2}:\left(x,y,0\right)\mapsto \left(x,y\right)$, the flattened membrane is a smooth manifold with the atlas {(*M*_0_, *π*)}.

In a next step, we regard dermal papillae as local evaginations from *M*_0_ in $R^{3}$, formalized as charts (*U*_*i*_, *φ*_*i*_) where the disjoint open sets $U_{i}\subset R^{3}$ describe the surfaces of nonoverlapping papillae and the images *φ*_*i*_(*U*_*i*_) = *V*_*i*_ are disjoint open disks in $R^{2}$. The planar part of the basal membrane between dermal papillae (compare rete ridges) can be formalized by $N_{0}:=M_{0}\backslash {⋃}_{i}\bar{\pi^{-1}\left(V_{i}\right)}$. The compatibility condition yields a differentiability requirement for the mappings *φ*_*i*_ and *π* at the interfaces between the papillae surfaces *U*_*i*_ and the planar membrane part *N*_0_. With {(*N*_0_, *π*)} ∪ {(*U*_*i*_, *φ*_*i*_)}_*i*_ we obtain an atlas for the (composite) manifold *M*_1_: = *N*_0_∪⋃_*i*_ *U*_*i*_ representing the basal membrane with protruding dermal papillae. Because *π* is the projection mapping, the representation of the basal membrane *M*_1_ in $R^{2}$ appears distorted only within the base areas of papillae *V*_*i*_.

In order to model coarser elevations and depressions, we additionally deform the membrane vertically by a mapping $\chi^{-1}:R^{3}\to R^{3}$. The final manifold surface *M*_2_: = *χ*^−1^(*M*_1_) is equipped with the atlas {(*χ*^−1^(*N*_0_), *π* ∘ *χ*)} ∪ {(*χ*^−1^(*U*_*i*_), *φ*_*i*_ ∘ *χ*)}_*i*_ and for convenience we introduce the notation $\psi :M_{2}\to R^{2}$ to refer to the collection of charts that identify locations on the basal membrane *M*_2_ with locations in $R^{2}$.

A key feature of the formalization by differentiable manifolds is the identification of tangential vectors on the vaulted basal membrane with tangential vectors in $R^{2}$ by the linear mapping

$$\begin{array}{c} d\psi_{x}:R^{3}⊃T_{x}M_{2}\to T_{\psi (x)}R^{2}=R^{2} \end{array}$$ (1)

where *x* ∈ *M*_2_ is a location on the manifold and *T*_*x*_ *M*_2_ is the tangent space at *x*. Secondly, geodesic paths on the membrane surface can be calculated by solving the geodesic differential equation in $R^{2}$. Both aspects are crucial for our modeling approach and allow to constrain cellular movement and diffusion to the basal layer. During iterative simulation small spatial displacements along the vaulted membrane are implemented as geodesic paths (S1 Text). For the corresponding algorithms to be computationally affordable, the mathematical formulation of the manifold must be analytically tractable as well as inexpensive and stable from a numerical perspective. The following section presents a geometric model for dermal papillae as charts (*U*_*i*_, *φ*_*i*_) or $\left(V_{i},\varphi_{i}^{-1}\right)$ that satisfies these requirements.

### Shape model for dermal papillae

We model each dermal papilla as a surface of revolution which is obtained by rotating a one-dimensional curve around a vertical axis in $R^{3}$. The parameterization of a dermal papilla is composed of height $H\in R_{+}$, base radius $R\in R_{+}$ and the shape curve *S*. According to the differentiability requirement, the surface of each papilla *U*_*i*_ must transition smoothly into the planar part *N*_0_ of the basement membrane. To meet this requirement and to be able to reproduce the characteristic lateral S-shape of dermal papillae, we model shape curves as three-times continuously differentiable paths,

$$\begin{array}{c} S:[0,R]\to [0,R]\times [0,H]:\theta \mapsto (r(\theta ),h(\theta )), \end{array}$$

where the curve parameter *θ* can be interpreted as a virtual radius. The components *r* and *h* are assumed to satisfy the boundary conditions

$$\begin{array}{cccc} r(0) & =0\; & r(R) & =R\\ r^{\prime }\left(0\right) & =1\; & r^{\prime }\left(R\right) & =1\\ r^{\left(j\right)}\left(0\right) & =0\; & r^{\left(j\right)}\left(R\right) & =0\\ h(0) & =H\; & h(R) & =0\\ h^{\left(k\right)}\left(0\right) & =0\; & h^{\left(k\right)}\left(R\right) & =0 \end{array}$$ (2)

for *j* = 2, 3 and *k* = 1, 2, 3.

With the parameter tuple (*x*_*i*_, *R*_*i*_, *H*_*i*_, *r*_*i*_, *h*_*i*_), where *R*_*i*_ is the radius of a disk *V*_*i*_ with center $x_{i}\in R^{2}$, each dermal papilla can be represented as a chart $\varphi_{i}^{-1}:V_{i}\to U_{i}$, with local polar representation

$$\begin{array}{c} \varphi_{i}^{-1}:\{\begin{array}{cc} \left[0,2\pi \right)\times \left[0,R_{i}\right] & \to R^{3}\\ \left(\alpha ,\theta \right) & \mapsto (r_{i}\left(\theta \right)\cos\alpha ,r_{i}\left(\theta \right)\sin\alpha ,h_{i}\left(\theta \right)) \end{array}. \end{array}$$

**Fig 7:**
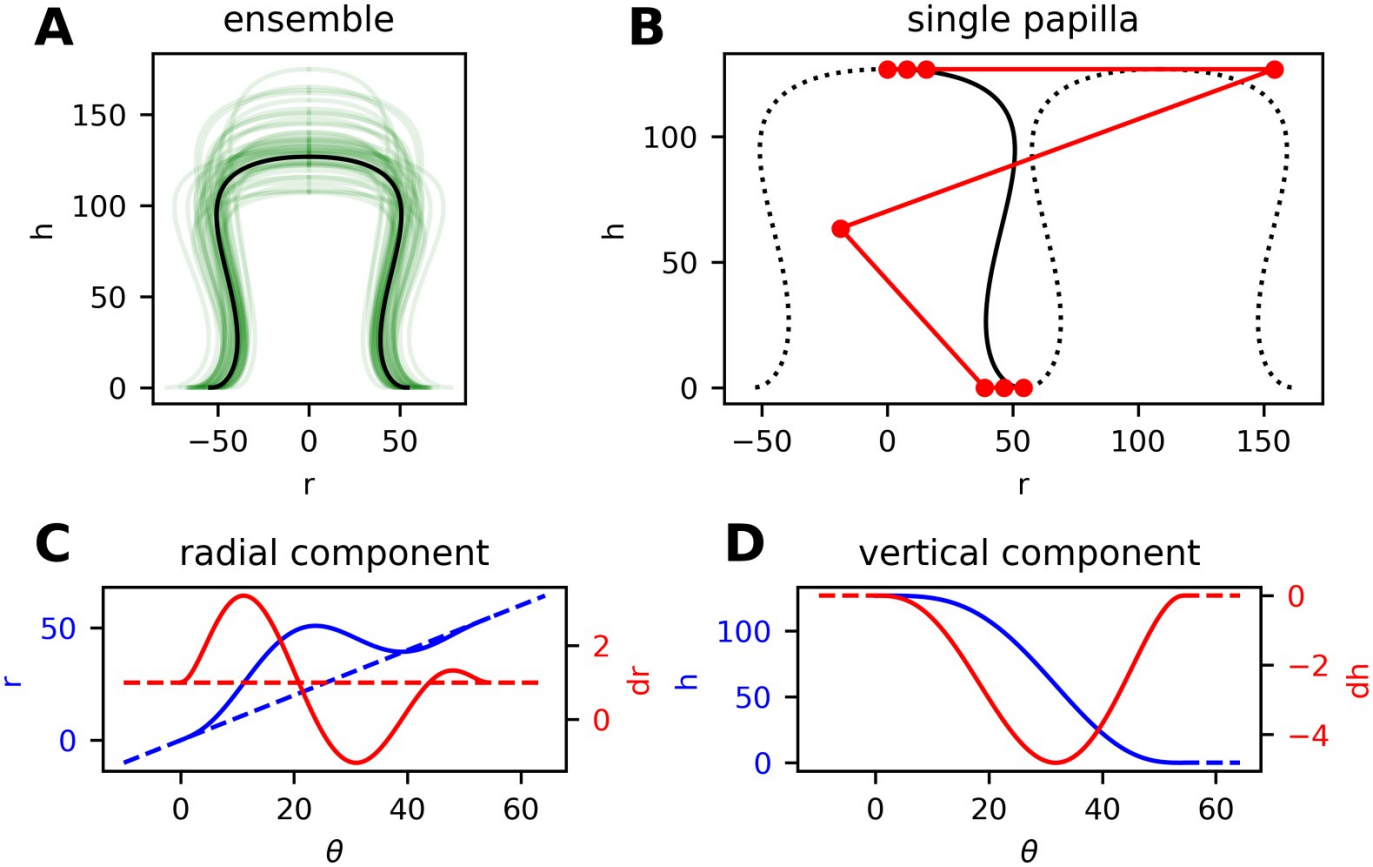
B-spline model for the shape of dermal papillae. (A) Lateral section of an ensemble of randomly generated papillae (green lines). (B) Positioning of control vertices (red markers connected by lines) for a particular shape curve (black). The positions of the fourth and fifth vertex along the *r*-axis can be altered with the shape parameter *p*. (C, D) Radial and vertical component of the shape curve (blue) with corresponding derivatives (red) and auxiliary lines. Compare the differentiability requirements in Eq (2).

We use a combined B-spline approach for the scalar components *r* and *h* such that the shape of dermal papillae can be modified by the displacement of individual control vertices via a single parameter *p*_*i*_ (Fig 7). Accordingly, the parameterization of a dermal papilla can be reduced to a tuple (*x*_*i*_, *R*_*i*_, *H*_*i*_, *p*_*i*_).

The transformation *χ*^−1^ is modeled as a superposition of larger circular elevations and depressions. For the vertical components the function model for *h* can be reused.

### Parameterization of the geometric model

We measured the dimensions of 100 dermal papillae from routine histopathologic sections of a retrospectively obtained convenience sample of 13 biopsies. Measured dimensions include the height $\bar{H}$, radius (half of the diameter) at the base ${\bar{r}}_{h=0}$, at half of the height ${\bar{r}}_{h=0.5H}$ and at 10 μm below the tip ${\bar{r}}_{h=H-10}$. The average measured height was 59.7 μm with a standard deviation of 18.2 and the mean base radius was 28.0 μm with a standard deviation of 10.1. The raw measurement data is provided in S1 Table. We accounted for dehydration by increasing all lengths by 20% [52] but still assumed a certain underestimation and distortion in the measurements due to inhomogeneous shrinking and deformation. Additionally, the histological section technique implies that papillae are usually not cut at their thickest position and that the sectional plane does not perfectly fit the vertical alignment of papillae.

We used optimization techniques to fit the mathematical model for shape curves (*R*, *H*, *p*) to each recorded papilla $\left(\bar{H},{\bar{r}}_{h=0},{\bar{r}}_{h=0.5H},{\bar{r}}_{h=H-10}\right)$ individually. Neglecting any statistical correlations, the solution ensemble (parameters *R* and *p*) as well as additional measurements were fitted with beta and log-normal distributions. Based on the resulting statistical model we were able to randomly generate dermal papillae and basal membranes.

From visual inspection and comparison with horizontal reflectance confocal microscopic images taken in vivo (compare Fig 2F and 2G), we came to the conclusion that simulation of realistic tissue anatomy requires approximately twice the radius (and hight) as we extracted from histopathologic measurements. Hence, artificial papillae are sampled with a base radius between 56 and 75 μm (standard deviation between 8.5 and 11) and a height between 70 and 135 μm (standard deviation between 13 and 21) depending on the simulated characteristics of skin tissue. We also recognized that the shape of measured papillae is equally distributed between the broad-shouldered and cylindrical types. In the parameterization of the geometric model we intentionally introduce an over-representation of the shouldered type (S-shape) in order to give enough space for the formation of melanocytic nests between (non-deformable) dermal papillae.

To validate the number of dermal papillae per skin area, we counted dermal papillae on 40 1 mm x 1 mm sectors of the inner lower arm of a 35-year male volunteer via horizontal in vivo reflectance confocal microscopy (VivaScope 3000; MAVIG GmbH, Munich, Germany; raw data in S1 Table). We found a mean of 48.8 papillae per mm^2^ with standard deviation of 5.29 corresponding to values reported in literature [68–71]. Due to the absence of additional skin features like eccrine gland ducts and hair follicles in silico, the density of papillae in the computer model must be higher in order to obtain realistic spacing (corresponding to the dimensions of rete ridges). Also the perfect circular contour of artificial papillae introduces artifacts in the perception of the density. We came to the conclusion that the optimal result is obtained with maximum packing density, corresponding to a value between 50 and 160 papillae per mm^2^ depending on the typical size of papillae and the simulated tissue characteristics. The model parameters obtained with this approach are presented in Table A in S2 Text.

From the papilla shape model (Fig 7) it follows that the largest diameter of dermal papillae is always attained at the base. As a consequence, the problem of generating dense spatial layouts of non-intersecting papilla bodies can be reduced to placing non-overlapping disks on a plane. To generate spatial layouts with maximum packing density, we align papillae on a hexagonal grid and use iterative local optimization of their center positions to account for the differences in disk radii.

### Melanocyte population dynamics

We developed an iterative scheme that advances the melanocyte-agents in constant time steps Δ*t*. A typical value for Δ*t* is 1 d, but all algorithms and calculations were designed to scale correctly with different time increments (see S6 Video and also S3 and S4 Text). To reduce the effect of the initial conditions and the warm-up phase (high stochastic volatility due to small population) on the overall long-term result, simulations are usually initialized with a group of about 10 melanocytes located at the center of the basal membrane segment.

According to our conceptual model, we assume that certain intra- and extracellular conditions can impact the rate of proliferation [16, 54]. Hence, individual cell behavior is regulated by two *agent-centric* state values of which the first is the generation number and the second is the local density. The generation number *g* is an integer value that counts the number of cell divisions a particular cell has undergone since the start of a simulation run. We define the local density as the distance-weighted neighborhood measure

$$\begin{array}{c} \rho =\sum_{n\in M}\omega \left(d_{n}\right) \end{array}$$

where *M* is the set of all melanocytes, *d*_*n*_ is the distance to a particular neighboring cell and *ω* is a weighting function. Most notably, this approach provides the necessary flexibility in giving nearby cells a greater impact on the local density than distant cells. Density values greater than one are clamped such that the measure can be interpreted as the ratio between the local number of melanocytes and the theoretical maximum configuration. We use a quadratically decaying weighting function *ω* taking a maximum value of 0.02 at zero distance and vanishing at a distance of 100 μm. This is of course a heuristic choice and the resulting density must be treated as a dimensionless value. The density measure is however not used to quantify the population but rather serves as an input signal for controlling individual cell behavior.

Overall, we expect exponential growth dynamics in the number of melanocytes $M^{\prime }\left(t\right)=\tilde{p}\;M\left(t\right)$ with the proliferation rate $\tilde{p}$ degrading with generation and density such that $\tilde{p}=\tilde{p}\left(g,\rho \right)=A\left(g\right)\;B\left(\rho \right)\;p_{0}$ where *A* and *B* are polynomial functions with values in the interval [0, 1] and *p*_0_ is the base proliferation rate. For instance, to limit the maximum allowed generation to 60, we let *A*(*g*)→0 with *g* → 60. The density-dependent damping factor *B*(*ρ*) is used to reduce population growth in already congested (high-density) areas. In the quantification of growth rates of melanocytic lesions in vivo different measures are possible [5, 72]. The deduction of cell-level proliferation rates from macroscopic data is difficult and we assume that division rates can vary greatly depending on environment, cell-type, and differentiation [33]. Experimental studies in vitro indicate values between 0.02 h^−1^ and 0.04 h^−1^ for melanoma cells [30, 36]. We heuristically set the base proliferation rate *p*_0_ to 0.05 d^−1^. To simulate elevated proliferation within melanocytic nests we use a proliferation rate *p*_0_ of 0.1 d^−1^. From the macroscopic exponential growth model we calculate the individual per-step cell-division probability

$$\begin{array}{c} p\left(g,\rho \right)=\exp(A\left(g\right)\;B\left(\rho \right)\;p_{0}\;\Delta t)-1\approx A\left(g\right)\;B\left(\rho \right)\;p_{0}\;\Delta t. \end{array}$$

Our model neglects programmed cell death (apoptosis), only cells leaving the simulation domain are discarded. Cell division is assumed to be symmetric; if a division occurs in a cell, the generation number of both filial cells is increased by one. Various individual attributes of the parent such as size, location but also the base proliferation rate *p*_0_ are inherited. This enables the simulation of ancestral strains of melanocytes with specific *genetic* expressions [13, 17, 29] (e.g. for nested melanocytes *p*_0_ = 0.1). We distort the inherited values with additive Gaussian noise (e.g. *σ* = 0.01 for the base proliferation rate). During division of normal cells, differentiation into the nesting type occurs with a (conditional) probability *q*_0_, which is set to 0 in scenarios with only the reticular pattern and 0.1 in globular scenarios. Nested cells are emitted with a base rate *s*_0_ of 0.1 d^−1^.

An overview on the parameterization of cell proliferation is presented in Table A in S2 Text. Visual outlines of the algorithms and routines as well as further implementation details are presented in S6 Text. Possible configurations of the generation dependent damping *A*(*g*) and an effects analysis in the aggregated exponential growth model can be found in S3 Text. A comparison of different within-nest proliferation dynamics can be found in S4 Text and S3 Video.

### Cell migration model

The following iterative algorithm is used to simulate the migration of cells. For each cell-agent a new position at time *t* + Δ*t* is calculated from the current position taking into account the cells generation, the local density, interactions and collisions with other cells and the physiological constraint. A visual schematic of the movement model was presented in Fig 3. Here, we present in detail the mathematical formulation of this model. An outline of the corresponding algorithm and implemented code is provided in S6 Text.

At the beginning of each movement phase, vectors pointing to neighboring cells *n* and the respective distances are calculated by

$$\begin{array}{ccc} v_{n} & =x_{n}^{\left(t\right)}-x^{\left(t\right)},\; & n\in M\\ d_{n} & =\|v_{n}\|-r_{n}-r,\; & n\in M \end{array}$$

where *x*^(*t*)^ indicates the position of the cell at time *t* and *r* is its radius (for neighboring cells respectively). The local melanocyte density is obtained from *ρ* = ∑_*n*∈*M*_ *ω*(*d*_*n*_).

An initial velocity vector *v*^(*i*)^ is composed of external forces *v*_ext_, such as a downwards moving trend, and diffusive noise. The noise term is a trivariate normal random variable scaled by the base diffusivity *D* and damping factors *Q* and *R*, such that

$$\begin{array}{c} v^{\left(i\right)}=v_{\text{ext}}+\sqrt{2\;D\;Q(\rho )\;R(g)}\;{\left(\Delta t\right)}^{-\frac{1}{2}}\;v_{\text{rand}}. \end{array}$$

We use base diffusivity values in the range *D* ∈ [0, 60] μm^2^ d^−1^ depending on the simulation scenario. Diffusivity values for cell motility reported in literature can vary in orders of magnitude, however, experiments in vitro and in silico indicate corresponding values [30, 36]. External forces can be, for instance, downwards forces with their intensity depending on the altitude of cells above the membrane surface. A strong external force is given by a vector with length in the range ‖*v*_ext_‖ = 0.5. As noted before, these values are dimensionless and heuristic choices.

For each neighboring cell *n*, attractive and repulsive forces can modulate the initial velocity. Depending on the distance to the neighbor, on local cell density and on the generation number, the functions *F*_*a*_ and *F*_*r*_ in combination with the functional damping factors *G*_a,r_ and *H*_a,r_ determine the orientation and intensity of pairwise intercellular force vectors (compare Fig 3A).

$$\begin{array}{c} v^{\left(ii\right)}=v^{\left(i\right)}+\sum_{n\in M}\frac{v_{n}}{\|v_{n}\|}\;(F_{a}\left(d_{n}\right)\;G_{a}\left(\rho \right)\;H_{a}\left(g\right)-F_{r}\left(d_{n}\right)\;G_{r}\left(\rho \right)\;H_{r}\left(g\right)) \end{array}$$

The damping functions G_*_ and H_*_ take again values in the interval [0, 1]. Intercellular forces typically take values below 1 and disappear with increasing distance. For instance, to simulate cell-cell adhesion within nests, we use strong attractive forces that degrade at medium distance, *F*_*a*_(0) = 1 and *F*_*a*_(*d*) = 0 for *d* > 50.

The following *corrector* mechanism implements collisions between cell-agents. If the predicted velocity vector either leads to a collision with a neighbor *n* or if prevailing overlap will not be resolved automatically ($d_{n}<\frac{v_{n}}{\|v_{n}\|}\cdot v^{\left(ii\right)}\;\Delta t$), a fraction *K* of the respective projected velocity component is subtracted from the predicted vector (compare Fig 3B).

$$\begin{array}{c} v^{\left(iii\right)}=v^{\left(ii\right)}-\frac{v_{n}}{\|v_{n}\|}\;(\frac{v_{n}}{\|v_{n}\|}\cdot v^{\left(ii\right)}\;\Delta t-d_{n})\;\frac{K}{\Delta t}\;\left(\forall n\;\text{formally}\right) \end{array}$$

The intention of this algorithm is to minimize the overlap of simulated cells to a certain degree but not to provide *physical* collisions among spherical objects and conservation of momentum. The latter is usually formulated in terms of Lagrangian mechanics and sophisticated numerical algorithms are used [73]. The presented approach can be regarded a strong simplification of such mechanical formulations. Similar approaches (under different technical terms) are widespread in the simulation of cell migration [23, 26, 27, 37]. Alternative abstracted movement models are used in lattice models for simulating cellular motility [29–31, 34, 36]. In all cases, the movement rules described by a mathematical model are strong abstractions and aggregations of biomechanical dynamics.

In order to confine cells to the basal layer (we assume the cell is currently located on the membrane surface, compare Fig 3C), the velocity vector *v*^(*iii*)^ is projected (or rotated) into the tangential plane of the manifold *M*_2_ at location *x*^(*t*)^. The resulting tangential vector *v*^(*iv*)^ is then transformed into a tangential vector $\bar{v}:=d\psi_{x^{\left(t\right)}}\cdot v^{\left(iv\right)}$ of the coordinate space $R^{2}$ at location ${\bar{x}}^{\left(t\right)}:=\psi \left(x^{\left(t\right)}\right)$ using the linear mapping in Eq (1). By solving the geodesic differential equation with initial conditions $\left({\bar{x}}^{\left(t\right)},\bar{v}\right)$ on the interval [*t*, *t* + Δ*t*], a new parameter location ${\bar{x}}^{\left(t+\Delta t\right)}\in R^{2}$ is obtained. Finally, the new position on the basal membrane *M*_2_ is the inverse transform $x^{\left(t+\Delta t\right)}=\psi^{-1}({\bar{x}}^{\left(t+\Delta t\right)})$. Further details on the application of the geodesic differential equation can be found in S1 Text.

For cells that are part of a nest we skip the last step and calculate the new position as *x*^(*t*+Δ*t*)^ = *x*^(*t*)^ + Δ*t*(*v*^(*iii*)^ + *v*_derm_) where *v*_derm_ is non-zero only if the cell is located in the dermis (below the membrane). For simplicity *v*_derm_ is either a vertical vector if the cell is below the base level of the membrane or a horizontal vector if the cell is located within a dermal papilla.

A parameter overview is presented in Table A in S2 Text. For simulations we always assume the default case with absent intercellular forces *F*_a,r_ ≡ 0 and constant damping factors (≡ 1).

### Visualization techniques

Dermatoscopic and histopathologic diagnosis methods are the current state-of-the-art for evaluation of melanocytic lesions. We developed rendering techniques that visualize the physiology and global status of simulated cell populations in ways mimicking the image styles of both microscopic techniques. Dermatoscopy, as a non-invasive screening technique [41, 42], enables physicians through cross-polarization or immersion fluid to have an *en-face* view of pigmented lesions on the dermo-epidermal junction. However, melanocytes are not directly visible, but morphological features of melanocytic lesions are indicated by the local pigmentation of epidermal tissue. To produce comparable visual representations, we calculate two-dimensional horizontally resolved histograms from the positioning of simulated melanocytes and apply Gaussian filters on the normalized data in order to approximate tissue pigmentation. As a consequence, all melanocytes are assumed to eradiate pigmentation in a radially symmetric fashion irrespective of microanatomic boundary conditions and depth. To obtain more realistic visual representations, we plan to use three-dimensional rendering techniques, where effects like the depth-dependent translucency of epidermal tissue, normalization of color-space and the basal membrane are taken into account. Histopathological analysis, on the other hand, is the current gold-standard for a final and confirmative diagnosis of skin tumors, albeit evaluation is not entirely independent from clinical information [74]. In typical hematoxylin and eosin stained biopsy samples, pigmentation can be recognized to occur mainly in keratinocytes; melanocytes are usually typified by a white halo around the nucleus. In the computer generated histologic image style we represent sliced melanocytes as brown disks.

## Supporting information

S1 Text Geodesic model for cellular migration. Excursus on the simulation of cellular migration by geodesic translations along the surface of dermal papillae. We present some mathematical and technical details and compare the time-average vertical distribution of simulated cells in different configurations of this modeling approach.
(PDF)

S2 Text Parameterization overview. Tabular overview on the parameterization of simulation scenarios presented in this paper.
(PDF)

S3 Text Global reproduction dynamics. A discussion of exponential growth dynamics in a scenario with unlimited resources and without cell-cell interaction. A corresponding difference equations model for the expected number of melanocytes is presented.
(PDF)

S4 Text Dynamic control of nest size. We present equations for the dynamic evolution of the size of nests and compare the resulting functions with the average size of simulated nests.
(PDF)

S5 Text Stochastic variability. We show that repeated simulation runs with the same parameterization produce visually and quantitatively similar results.
(PDF)

S6 Text Programming and implementation. This note contains a short discussion on the mathematical formulation and implementation of simulation models in general. We further present the structural outline of our implementation and include excerpts of the core components of our code.
(PDF)

S1 Video Test of the differential geometric movement model. The video compares three different models for the movement of cells along the vaulted basal membrane. Left: Scenario 1 shows diffusive random motion with an additional downwards force. Center: Scenario 2 shows diffusive motion without downwards force. Right: Scenario 3 shows a fictional modification where cells maintain their inertia. In each scenario, seven cell-agents are simulated; cells do not divide and collisions are ignored. Color encodes altitude above the base of the rete ridges (blue: 0 μm, red: 200 μm). The simulated tissue segment measures 1,000 μm × 1,000 μm. In the top, the value indicated by T displays the simulation time measured in days.
(MP4)

S2 Video Temporal evolution of a simulated reticular nevus. This video displays the growth phase of a reticular nevus in different visual styles. Left: Spatial configuration of simulated cells with color indicating the cell generation number (blue: 0, red: 60). Right: Dermatoscopic visualization. Bottom: Vertical histologic view. Parameterization corresponds to scenario 4 in Fig 4. The simulated tissue segment measures 5,000 μm × 5,000 μm. In the top left corner, the values indicated by F, T and M display the frame number, the simulation time measured in days and the number of simulated melanocytes.
(MP4)

S3 Video Simulation of single nests. Side-by-side visualization of the evolution of four single nests (top: horizontal view, bottom: histologic view). The individual parameter configurations correspond to the four scenarios in Fig 5. Each nest starts in the center of a 1,000 μm × 1,000 μm tissue section. Cells that are part of the nest are displayed in light blue; emitted cells are displayed in dark blue. Emitted cells do not proliferate.
(MP4)

S4 Video Temporal evolution of a dotted nevus. The video displays the spatial evolution of a dotted nevus in four different visual styles as well as in the vertical histologic perspective. Top left: Color encodes generation number (blue: 0, red: 60). Top right: Dermatoscopic visualization. Center left: A random number of ancestral strains displayed in different colors. Center right: Nested and stray cells encoded in different color. Bottom: Histologic view. The model parameterization corresponds to scenario 1 in Fig 5. The simulated tissue segment measures 5,000 μm × 5,000 μm. In the top left corner, the values indicated by F, T and M display the frame number, the simulation time measured in days and the number of simulated melanocytes.
(MP4)

S5 Video Temporal evolution of a peripherally dotted nevus. The video displays the spatial evolution of a peripherally dotted nevus in four different visual styles as well as in the vertical histologic perspective. Top left: Color encodes generation number (blue: 0, red: 60). Top right: Dermatoscopic visualization. Center left: A random number of ancestral strains displayed in different colors. Center right: Nested and stray cells encoded in different color. Bottom: Histologic view. The model parameterization corresponds to scenario 4 in Fig 5. The simulated tissue segment measures 5,000 μm × 5,000 μm. In the top left corner, the values indicated by F, T and M display the frame number, the simulation time measured in days and the number of simulated melanocytes.
(MP4)

S6 Video Reticular nevus simulated with different time increments. Side-by-side comparison of two simulation runs (reticular nevus) with identical parameterization but different time increments. Left: Δ*t* = 0.1 d. Right: Δ*t* = 1.0 d. Top: Dermatoscopic visualization. Center: Altitude of cells above the base of the rete ridges encoded in color (blue: 0 μm, red: 200 μm). Bottom: Vertical histologic views. The image side lengths correspond to 1,000 μm. In the top, the values indicated by F, T and M display the frame number, the simulation time measured in days and the number of simulated melanocytes. Note that the different frame rates (Δ*t*) result in a different frame count.
(MP4)

S1 Table Raw microanatomic measurement data. Measured in histopathologic images: melanocyte diameters, dimensions of dermal papillae and rete ridges. Measured via confocal laser scanning microscopy: density of dermal papillae per skin area.
(XLSX)

S2 Table Growth data obtained in vivo from dermatoscopic images. This data was obtained from retrospectively collected sets of anonymized dermatoscopic images. The area of nevi was determined by image segmentation with a neural network.
(XLSX)
