# Supplementary material for: Reproduction of patterns in melanocytic proliferations by agent-based simulation and geometric modeling
Source: PLoS Comput Biol. 2021 Feb 4;17(2):e1008660. doi: 10.1371/journal.pcbi.1008660 (PMC7888658; doi:10.1371/journal.pcbi.1008660)
Supplement: S1 Text — Excursus on the simulation of cellular migration by geodesic translations along the surface of dermal papillae. We present some mathematical and technical details and compare the time-average vertical distribution of simulated cells in different configurations of this modeling approach. (PDF) [file pcbi.1008660.s001.pdf]

S1 Text: Geodesic model for cellular migration

We simulate the motion of cells along the surface of the basal membrane as geodesic paths. This allows to confine cells in the basal layer and, secondly, yields homogeneous diffusion behavior on the surface space.

The unique geodesic path for a given initial location $x(t)$ and velocity $v(t)$ on the manifold M_1 can be obtained from the solution of the geodesic equation. Let $c : [t, t + \Delta t] \to \mathbb{R}^2$ be a path in the coordinate space that solves the nonlinear ordinary differential equation

$$\ddot{c}_k + \sum_{i,j=1}^{2} \Gamma_{ij}^{k} \, \dot{c}_i \, \dot{c}_j = 0 \qquad k = 1, 2$$

with initial conditions $c(t) = \psi(x(t))$ and $\dot{c}(t) = d\psi_{x(t)} \cdot v(t)$ where Γ_{ij}^{k} are the Christoffel symbols of the manifold. Then $\psi^{-1} \circ c : [t, t + \Delta t] \to M_2$ is the unique geodesic path on M_1.

Because we use local polar coordinate representations $(\alpha, \theta) \in [0, 2\pi) \times [0, R_i]$ for dermal papillae, we switch between geodesic paths in N_0, which are essentially straight lines, and geodesic paths of the form

$$\tau \mapsto \Big(x_i + r\big(\theta(\tau)\big) \cos \alpha(\tau), \; y_i + r\big(\theta(\tau)\big) \sin \alpha(\tau), \; h\big(\theta(\tau)\big) \Big)$$

on U_i where (x_i, y_i) is the center location of the dermal papilla. In a small flat top area of papillae we switch back to Cartesian coordinates in order to avoid numerical issues when solving the differential equations.

In Figure A and in S1 Video we compare diffusive migration of cells with (scenario 1) and without (scenario 2) downwards trend and on geodesic trajectories resulting from the conservation of inertia (scenario 3). Scenarios 1 and 2 reproduce the inhomogeneous vertical distribution of melanocytes often found in histologic images of melanocytic lesions. Scenario 3 demonstrates the technical implementation of the geodesic movement model.

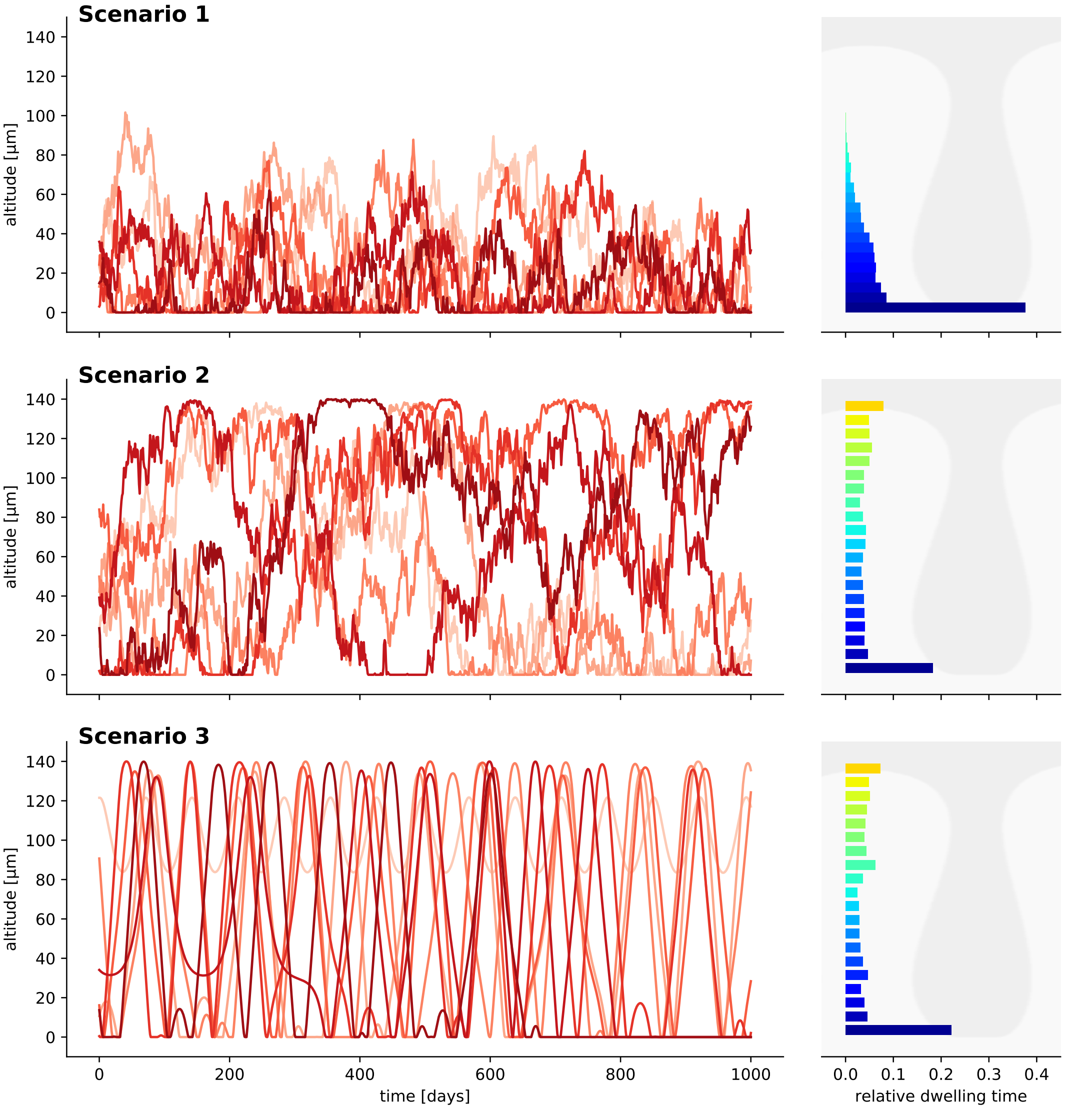

Figure A. Vertical distribution of cell agents. To facilitate comparison, a tissue segment with equally high dermal papillae (140 µm) was generated. The altitude of seven cell agents with locally diffusive motion (scenarios 1 and 2, with and without a downwards force field) and with constant velocity geodesic trajectories (scenario 3) was tracked (red lines in different color intensity). Collisions and density effects were neglected, no cell divisions were allowed. On the right, histograms show the relative dwelling time in different horizontal layers of the dermo-epidermal junction. In S1 Video, the same trajectories are visualized from a horizontal perspective.
