# Supplementary material for: Reproduction of patterns in melanocytic proliferations by agent-based simulation and geometric modeling
Source: PLoS Comput Biol. 2021 Feb 4;17(2):e1008660. doi: 10.1371/journal.pcbi.1008660 (PMC7888658; doi:10.1371/journal.pcbi.1008660)
Supplement: S2 Text — Tabular overview on the parameterization of simulation scenarios presented in this paper. (PDF) [file pcbi.1008660.s002.pdf]

S2 Text: Parameterization overview

To model functional parameters such as variable damping factors or spatial influences we use smoothstep functions, other polynomial functions or combinations thereof. We introduce the notation $(a, b, c) \mapsto (x, y, z)$ for a function with a single argument and a smooth transition between the input values $a < b < c$ and the function values x, y, z. We assume that for arguments smaller than a, the function takes the first value x. When the argument approaches the intermediate value b, the function value approaches y. At c or above, the function value is z.

The parameter values in the following tables are discussed and referenced in *Methods* in the main text.

Table A. Overview on model variables and parameters. According to the construction of the model, some parameters are constant values and others are subject to random noise and dynamic adaption during simulation. The numerical values shown represent a standard model configuration.

Variable name	Symbol	Value/Range	Unit
papillae radius [1]		$[50, 80]$<br>e.g. $\mathrm{LogNorm}(4.0, 0.15)$	µm
papillae height [1]		$[70, 140]$<br>e.g. $\mathrm{LogNorm}(4.9, 0.1)$	µm
papillae density [1,2]		$[50, 160]$	papillae per mm^2
density weight function [3]	$\omega(d)$	$(0, 100) \mapsto (0.02, 0)$	
time step	Δt	≤ 1	d
cell radius [4]	r	6 ± 0.7<br>$\mathrm{LogNorm}(1.8, 0.11)$	µm
local density [4]	ρ	$[0, 1]$	cells per volume
generation number [4]	g	$\mathbb{N}$	number of divisions
position [4]	x	$\mathbb{R}^3$	µm
base diffusivity [4,5]	D	$[0, 60]$	µm^2 d^{-1}
base proliferation rate [4,5]	p_0	0.05	d^{-1}
nest formation probability [5]	q_0	$[0, 0.1]$	per cell division
within nest proliferation rate [5]	p_0	0.1	d^{-1}
emission from nests [5]	s_0	0.1	d^{-1}

[1] Crude range, varies with the tissue model (geometric parameterization).
[2] Is not a configurable parameter, results from dense packing of papillae.
[3] Is a heuristic choice, we use a quadratically decaying function.
[4] Microscopic parameterization and state, varies across cells and is inherited during division.
[5] Modulated by state variables and the local configuration of the melanocyte population according to control functions.

Table B. Parameterization of reticular nevi. The model parameterizations in this table (Scenario 1-4) correspond to the presented scenarios in Fig 4 in the main text.

		Scenario 1	Scenario 2	Scenario 3	Scenario 4
base proliferation rate	p_0	0.05			
generation damping factor	$A(g)$	$(40, 60) \mapsto (1, 0)$			
density damping factor	$B(\rho)$	$(0.1, 0.25) \mapsto (1, 0)$	$(0.1, 0.3) \mapsto (1, 0)$	$(0.2, 0.5) \mapsto (1, 0)$	$(0.1, 0.3, 0.5, 1.0) \mapsto (1, 0.05, 0.05, 0.01)$
base diffusivity	D	30.0			
generation damping factor	$R(g)$	$(40, 60) \mapsto (1, 0.1)$		1	$(40, 60) \mapsto (1, 0)$
density damping factor	$Q(\rho)$	$(0.4, 0.8) \mapsto (1, 0.1)$	$(0.2, 0.8) \mapsto (1, 0.1)$	$(0.5, 1) \mapsto (1, 0.1)$	$(0.4, 0.8) \mapsto (1, 0.05)$
external downwards force [1]		no	yes	yes	yes
mean papillae diameter [2]		small	large	small	large

[1] If applied, external downwards force depends on the vertical distance between the cell and the membrane (altitude) and is a smooth transition from 0 when the cell is located at the membrane to a maximum of 0.2 when the cell is located 200 µm or higher above the membrane. In short notation this can be written as $(0, 200) \mapsto (0, 0.2)$.

[2] The microanatomy is either characterized by small or large dermal papillae. In the first case the radius and height are randomly distributed with Log-normal distributions with logarithmic mean 4.0 and standard deviation 0.15 for the radius and logarithmic mean 4.9 and standard deviation 0.1 for the height. For the geometric configuration with larger dermal papillae the parameters are 4.3/0.15 and 4.9/0.1 respectively.

Table C. Parameterization of globular nevi. The model parameterizations in this table (Scenario 1-4) correspond to the presented scenarios in Fig 5 of the main text. Also compare the scenarios in S4 Text and the animations of single nests in S3 Video. Note that occasionally the same variable names are used for damping factors of stray cells and of nested cells. Damping factors not listed take the constant value 1. Forces not explicitly listed are absent.

<table>
<tr><th></th><th></th><th>Scenario 1</th><th>Scenario 2</th><th>Scenario 3</th><th>Scenario 4</th></tr>
<tr><td>base proliferation rate</td><td>p_0</td><td colspan="4">0.05</td></tr>
<tr><td>generation damping factor</td><td>$A(g)$</td><td colspan="4">$(40, 60) \mapsto (1, 0)$</td></tr>
<tr><td>density damping factor</td><td>$B(\rho)$</td><td colspan="4">$(0, 0.2) \mapsto (1, 0)$</td></tr>
<tr><td>base diffusivity</td><td>D</td><td colspan="4">40.0</td></tr>
<tr><td>generation damping factor</td><td>$R(g)$</td><td colspan="4">1</td></tr>
<tr><td>density damping factor</td><td>$Q(\rho)$</td><td colspan="4">$(0, 1) \mapsto (1, 0.3)$</td></tr>
<tr><td>external downwards force</td><td></td><td colspan="4">$(0, 200) \mapsto (0, 0.5)$</td></tr>
<tr><td>external downwards force in nests</td><td></td><td colspan="4">$(0, 50) \mapsto (0, 0.5)$</td></tr>
<tr><td>nest formation probability [1]</td><td>q_0</td><td>0.05</td><td>0.1</td><td>0.05</td><td>0.1</td></tr>
<tr><td>generation damping factor</td><td></td><td colspan="4">1</td></tr>
<tr><td>density damping factor</td><td></td><td colspan="4">$(0.2, 0.3) \mapsto (1, 0)$</td></tr>
<tr><td>base within-nest proliferation rate</td><td>p_0</td><td colspan="4">0.1</td></tr>
<tr><td>generation damping factor</td><td>$U(g)$</td><td>$(7, 8) \mapsto (1, 0)$</td><td colspan="2">$(6, 9) \mapsto (1, 0)$</td><td>$(5, 8) \mapsto (1, 0)$</td></tr>
<tr><td>density damping factor</td><td></td><td colspan="4">1</td></tr>
<tr><td>base nest emigration probability</td><td>s_0</td><td>0</td><td colspan="3">0.1</td></tr>
<tr><td>generation damping factor</td><td>$V(g)$</td><td>–</td><td>$(5, 7, 9) \mapsto (0, 0.45, 0)$</td><td>$(4, 18) \mapsto (0, 0.5)$</td><td>$(7, 10) \mapsto (0, 1)$</td></tr>
<tr><td>density damping factor</td><td></td><td>–</td><td colspan="3">1</td></tr>
<tr><td>base within-nest diffusivity</td><td>D</td><td colspan="4">0</td></tr>
<tr><td>intra-nest attraction distance [2]</td><td>$F_a(d)$</td><td colspan="4">$(0, 50) \mapsto (1, 0)$</td></tr>
<tr><td>inter-nest repulsion distance [2]</td><td>$F_r(d)$</td><td colspan="4">$(0, 10) \mapsto (0.5, 0)$</td></tr>
<tr><td>collective repulsion distance [2]</td><td>$F_r(d)$</td><td colspan="4">$(0, 20) \mapsto (1, 0)$</td></tr>
<tr><td>mean papillae diameter [3]</td><td></td><td>large</td><td>small</td><td>large</td><td>small</td></tr>
</table>

[1] Is a conditional probability given a cell division occurs.
[2] The same symbol is used for pairs of cells within the same nest, for pairs of cells in different nests and for pairs of stray cells and nested cells.
[3] The configuration of the microanatomy is analogous to Table B.
