# Supplementary material for: Reproduction of patterns in melanocytic proliferations by agent-based simulation and geometric modeling
Source: PLoS Comput Biol. 2021 Feb 4;17(2):e1008660. doi: 10.1371/journal.pcbi.1008660 (PMC7888658; doi:10.1371/journal.pcbi.1008660)
Supplement: S3 Text — A discussion of exponential growth dynamics in a scenario with unlimited resources and without cell-cell interaction. A corresponding difference equations model for the expected number of melanocytes is presented. (PDF) [file pcbi.1008660.s003.pdf]

S3 Text: Global reproduction dynamics

In a scenario with unlimited resources (i.e. space) growth of the melanocyte population is not influenced by cell-cell interaction ($B(\rho) \equiv 1$) but only limited by the generation dependent factor $A(g)$. According to the stochastic inheritance of the base proliferation rate, the individual probability for cell division can be approximated by

$$p(g) \approx \exp\Big(A(g)\,\big(p_0 + \sqrt{g}\sigma X\big)\,\Delta t\Big) - 1, \tag{1}$$

where σ is the strength of the noise and X is a standard normal random variable.

In the sample average, the approximation $\bar{p}(g) = \exp\big(A(g)\,p_0\,\Delta t\big) - 1$ is valid. The average population growth (mean-field model) can thus be formalized with the difference equations system

$$\begin{aligned} M_0(t+\Delta t) &= \big(1-\bar{p}(0)\big)\,M_0(t), \\ M_g(t+\Delta t) &= \big(1-\bar{p}(g)\big)\,M_g(t) + 2\,\bar{p}(g-1)\,M_{g-1}(t) \quad g = 1,2,\dots. \end{aligned} \tag{2}$$

In Figure A, results obtained from the approximate stochastic model (1) and the difference equation model (2) are presented. The results indicate qualitative characteristics of the growth dynamics of the cellular population in the agent-based model and are a generous quantitative upper bound for the population size.

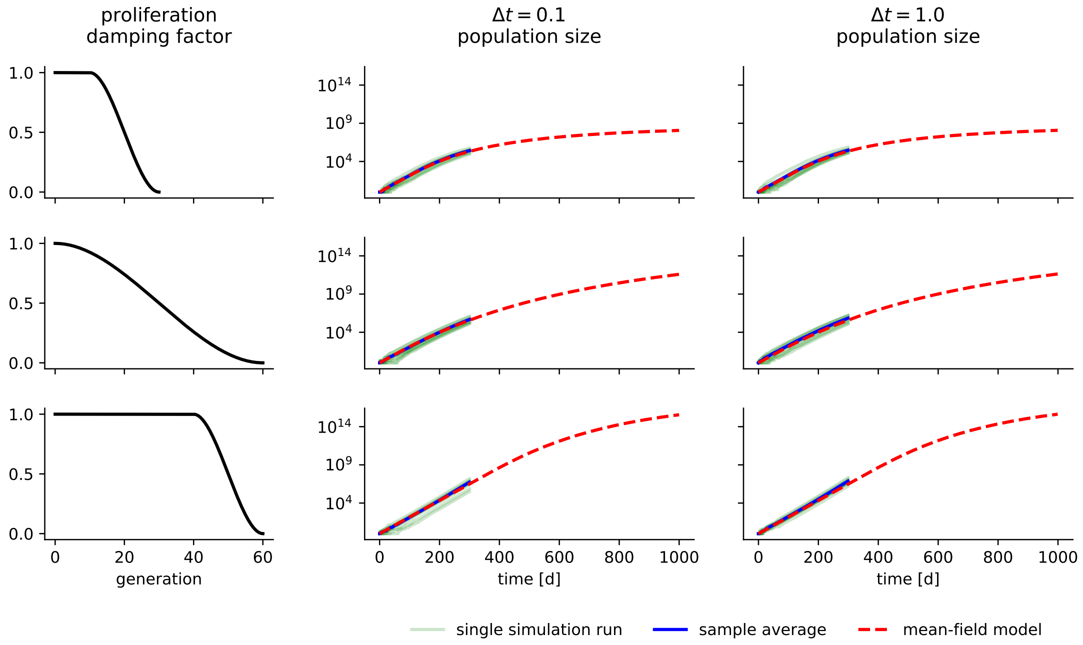

Figure A. Proliferation with unlimited resources. Damping of the reproduction probability in high-density regions is neglected so that the full proliferative potential can be exhausted. Different configurations of the generation dependent damping factor $A(g)$ are shown in the left column. For $\Delta t = 0.1$ and $\Delta t = 1.0$ corresponding solutions of the difference equations model (2) (red dashed) and 10 samples from the approximate individual based stochastic model (1) (green transparent) as well as the sample average (blue solid) are shown on the right. The stochastic simulation was limited to 300 days. The base reproduction probability was $p_0 = 0.05$ and the noise level was $\sigma = 0.01$.
