# Supplementary material for: Reproduction of patterns in melanocytic proliferations by agent-based simulation and geometric modeling
Source: PLoS Comput Biol. 2021 Feb 4;17(2):e1008660. doi: 10.1371/journal.pcbi.1008660 (PMC7888658; doi:10.1371/journal.pcbi.1008660)
Supplement: S4 Text — We present equations for the dynamic evolution of the size of nests and compare the resulting functions with the average size of simulated nests. (PDF) [file pcbi.1008660.s004.pdf]

S4 Text: Dynamic control of nest size

In the agent-based model, no intercellular influences, stochastic variation or inheritance was assumed for the within-nest reproduction and emission likelihoods. As a consequence the reproduction and emission probabilities can be written as

$$p(g) = \exp\big(U(g)\, p_0\, \Delta t\big) - 1,$$
$$s(g) = \exp\big(V(g)\, s_0\, \Delta t\big) - 1, \qquad (1)$$

where U and V are generation-dependent damping factors.

Let $N(t)$ describe the number of melanocytes in a particular nest and let $N_g(t)$ describe the subpopulation with generation number g. Clearly $N(t) = \sum_g N_g(t)$, such that the system of difference equations

$$N_0(t + \Delta t) = \big(1 - p(0) - s(0)\big) N_0(t),$$
$$N_g(t + \Delta t) = \big(1 - p(g) - s(g)\big) N_g(t) + 2\, p(g-1)\, N_{g-1}(t) \quad g = 1, 2, \ldots \qquad (2)$$

formalizes the average dynamic evolution of the size of a nest (mean-field model).

In contrast to the simulation of global population size with unlimited resources in S3 Text, here, the evolution of nest sizes is also a valid quantitative approximation. Figure A shows different configurations for persistent and dissolving nests and corresponding approximations with the model in equation (2) and stochastic simulation according to (1).

Visual animations of single nests with the configurations shown in the figure are available in S3 Video.

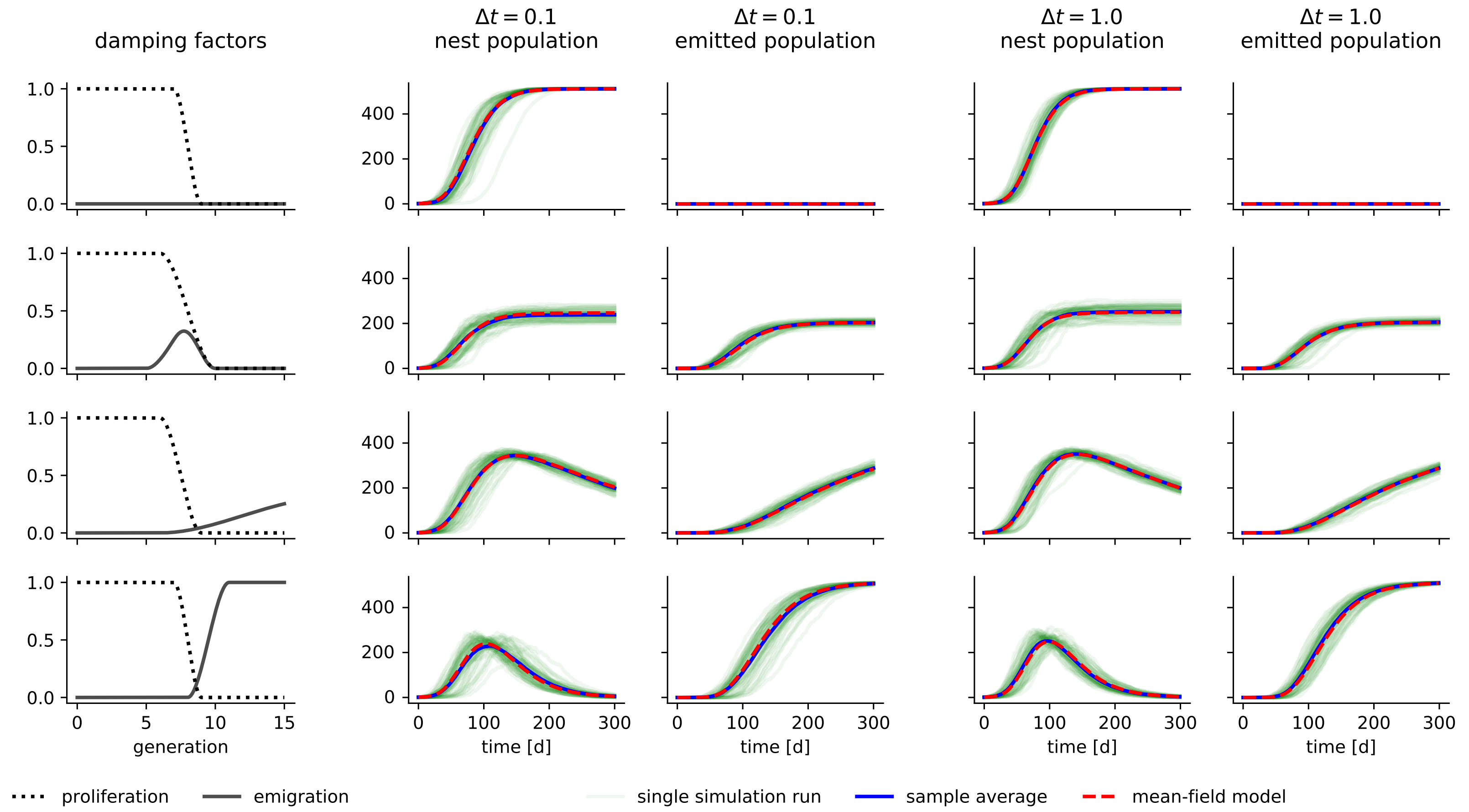

Figure A. Dynamic control of nest size. In the left column different configurations of within-nest proliferation (dotted) and emigration (solid) are shown. On the right, the resulting nest size and number of emitted cells from stochastic simulation (1) with 50 samples (transparent green) and the sample average (solid blue) as well as the difference equation model (2) (red dashed) are compared. Results are shown for different time steps Δt. The first scenario simulates persistent nests without emigration; in the second scenario a short period with emigration was added; the third and fourth scenario simulate volatile nests. Persistent nests occur when proliferation and emigration reach an equilibrium or when the emigration likelihood fades before proliferation is halted. For dissolving nests, it is necessary that the number of cells emigrating from the nest outweighs proliferative activity, at least in higher generations.
