# Supplementary material for: Reproduction of patterns in melanocytic proliferations by agent-based simulation and geometric modeling
Source: PLoS Comput Biol. 2021 Feb 4;17(2):e1008660. doi: 10.1371/journal.pcbi.1008660 (PMC7888658; doi:10.1371/journal.pcbi.1008660)
Supplement: S5 Text — We show that repeated simulation runs with the same parameterization produce visually and quantitatively similar results. (PDF) [file pcbi.1008660.s005.pdf]

S5 Text: Stochastic variability

Figures A and B show dermatoscopic visualizations and the temporal evolution of the population number of five simulation runs with the reticular and the globular pattern. In each group, the simulation runs were performed with the same model parameterization. Quantitative and qualitative differences (stochastic variability) are within reasonable bounds.

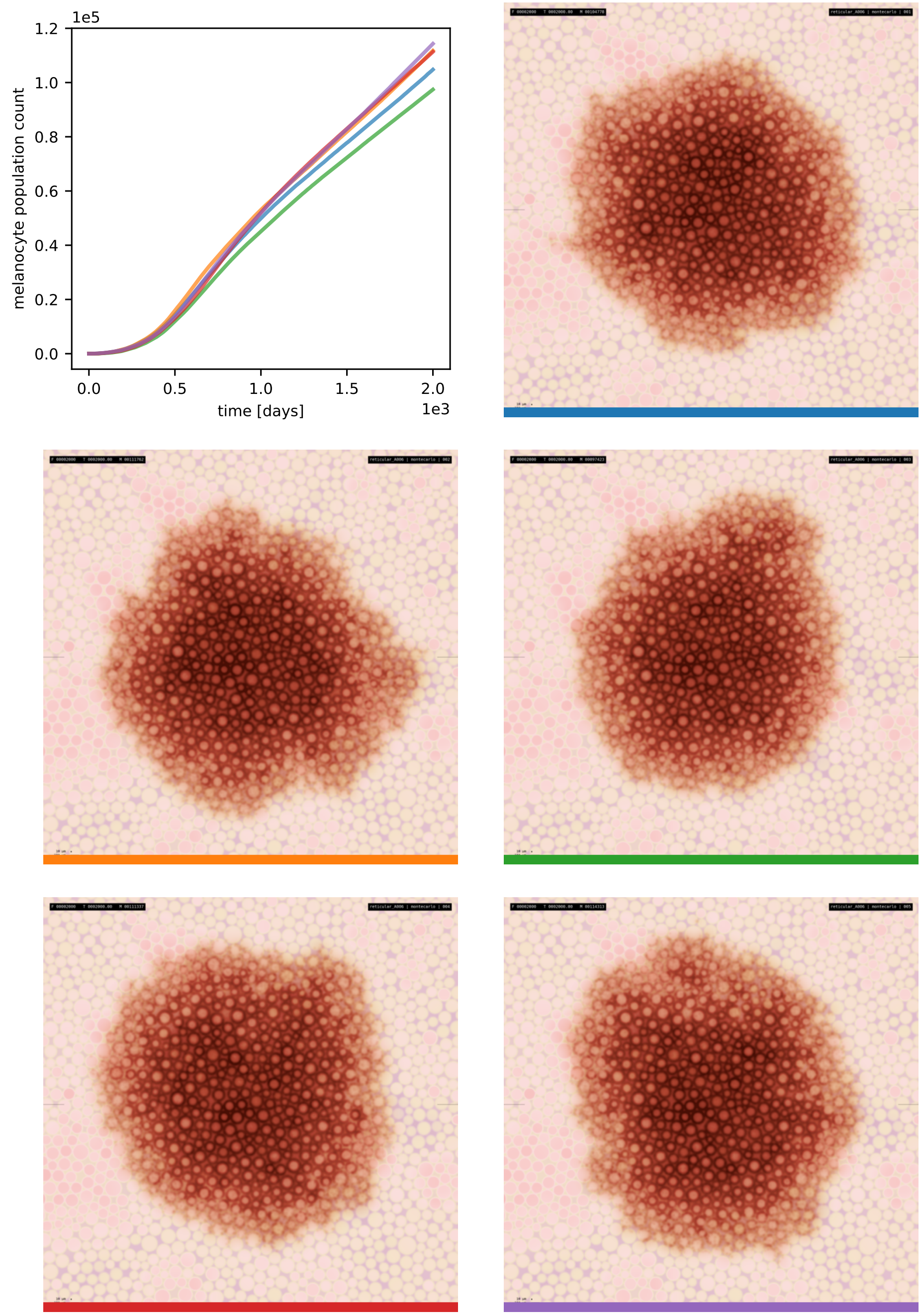

Figure A. Five simulation runs with a reticular configuration.

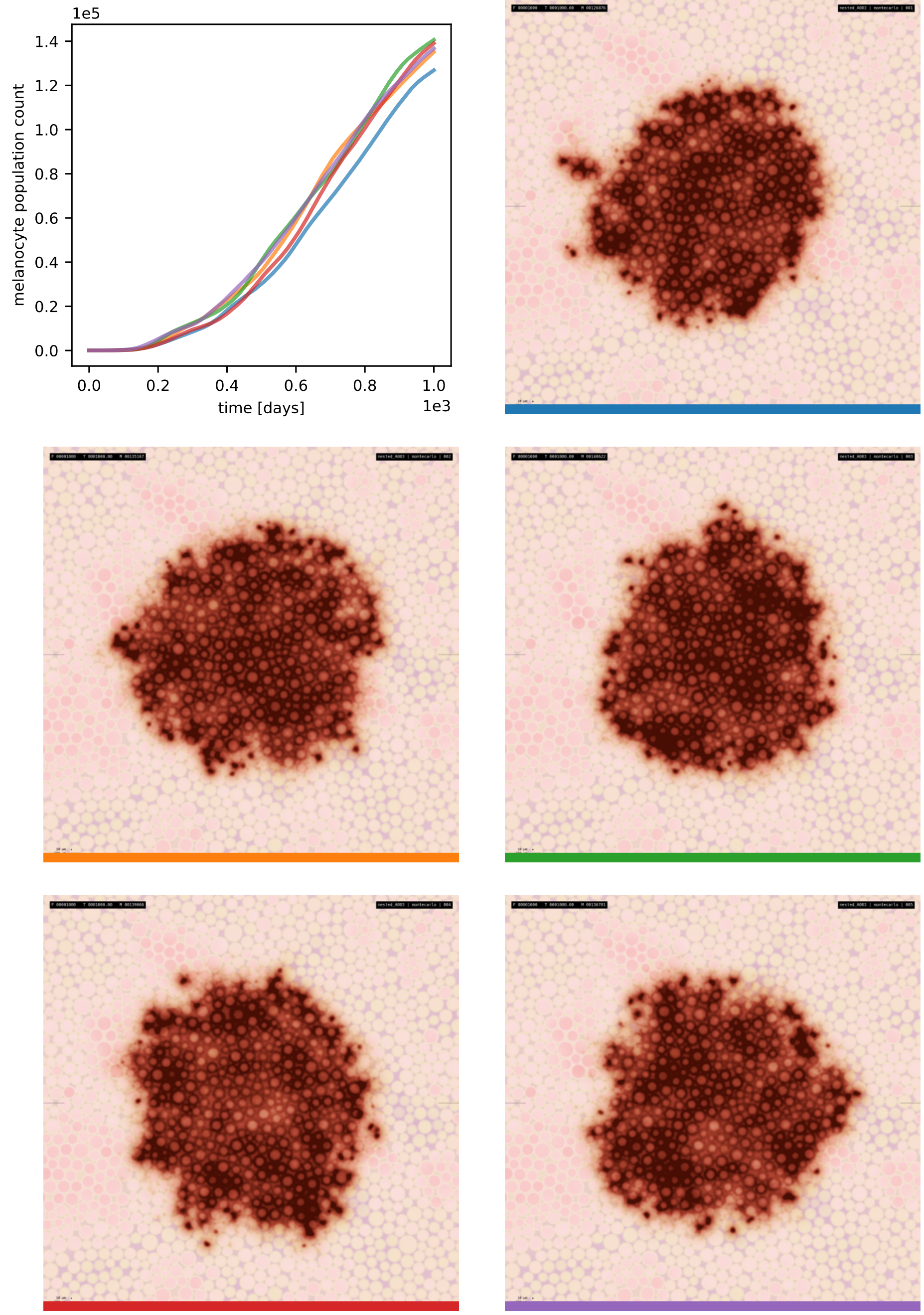

Figure B. Five simulation runs with a globular configuration.
