# Supplementary material for: Reproduction of patterns in melanocytic proliferations by agent-based simulation and geometric modeling
Source: PLoS Comput Biol. 2021 Feb 4;17(2):e1008660. doi: 10.1371/journal.pcbi.1008660 (PMC7888658; doi:10.1371/journal.pcbi.1008660)
Supplement: S6 Text — This note contains a short discussion on the mathematical formulation and implementation of simulation models in general. We further present the structural outline of our implementation and include excerpts of the core components of our code. (PDF) [file pcbi.1008660.s006.pdf]

S6 Text: Programming and implementation

This supporting text provides additional insight into our simulation approach on the implementation level.

A simulation model is constructed in several steps. Starting from an abstraction of a natural system and of the assumed dynamic processes, a conceptual model is constructed. Then this concept is formalized in terms of a mathematical model, which may consist of mathematical objects such as vectors or maps (state) and functional relations (evolution). The mathematical model is finally implemented in a programming environment, with the aim to solve or approximate the developed equations and routines.

We provide for our approach a clear motivation and discussion of the assumptions and abstractions that we made during conceptualizing the dermal-epidermal microphysiology and the behavior of melanocytes on a cellular scale (*Results*). We also compiled an exhaustive description of the associated mathematical models (*Methods*) and of the algorithmic outline of our simulator. In the following, we provide an overview on our implementation, visualize the logic structure of our code and present the programming of core components of our simulator.

Outline of simulation framework, program logic and algorithms

We present in this paper a simulation approach that combines a static geometric model of the basement membrane with an abstracted dynamic model of individual cell behavior. We compare our simulation results with clinical images by visualizing the global state of simulated cell populations. This setup is reflected in the structure of our implementation, which consists of three interconnected modules:

Geometry Stochastic generation of the microscopic anatomy of the dermal-epidermal junction. Implementation of the geometric transformations and methods for simulating cell-matrix interaction and constrained cellular movement.

Simulation Iterative scheme for simulating the temporal evolution of the melanocyte population. Stochastic simulation of cellular behavior such as division and nesting, calculation of individual movement vectors.

Visualization Data analysis and rendering techniques for generating statistical evaluations and imagery.

The simulation of a specific *scenario* begins with the definition of microanatomic (geometric) parameters and of intra- and inter-cellular model parameters. The former concerns the size of the simulation domain and the configuration of dermal papillae such as number, size and shape. The latter contains stochastic likelihoods for intracellular events (division, differentiation) and intercellular influences and forces (movement). All parameters are stored in configuration files on a per-scenario basis. The geometry module samples a random basal membrane instance and pre-renders visual representations that are required for the dermatoscopic and histopathologic visualization styles. To minimize computation time, instances of the geometric model can be reused for different simulation runs. The simulator initializes about 10 melanocytes in the center of the basal membrane segment. For every time step all melanocytes are processed individually (in parallel). This includes the calculation of the local density, intercellular force vectors (Figure A) and the simulation of stochastic events (Figure B). For simulating constrained movement in the basal layer, the calculated velocity vectors are transferred to the geometry module in order to obtain a new position on the membrane. Upon cell division, a copy of the parent is generated and the intracellular parameters of both resulting cells (symmetric division) are modified slightly. When the required number of time steps is reached, the visualization module is used to process the collected data, consisting of the configuration of each individual cell across all time steps. The following pseudo-code (Listing A) provides an algorithmic overview of this simulation routine.

Listing A. Pseudo-code outlining the algorithmic procedure for simulating a nevus with the implemented simulation framework.

```
GEOMETRY MODULE
1. load configuration file (ini)
2. generate Papillae layout
   a) sample individual shape parameters
   b) position papillae on the domain
3. store generated data (csv)
4. generate look-up-tables on disk (numpy data)
5. pre-render vertical and horizontal background images (png)

SIMULATION MODULE
1. load configuration file (ini) and geometry (csv, numpy data)
2. generate initial population of melanocytes
3. for each time step
      for each melanocyte
         a) movement
              calculate movement/velocity vector
                  - find all neighboring cells
                  - calculate vectors
                  - calculate local density
                  - calculate intercellular force vectors
                  - calculate external and random forces
              resolve collisions
              instantiate local geometry (membrane geometry, transformations)
              if not in nest
                 - transform velocity to 2D parameter space
                 - solve geodesic differential equation
                 - transform resulting position back to 3D space
              if in nest
                 - apply velocity on position in 3D
              displace cell accordingly

        b) intracellular events
             generate random numbers and process stochastic events
             if division
                 - if not in nest, allow to form new nest
                 - create a copy of the cell
                 - add random noise to certain state variables of both cells
             if not division
                 - if in nest, allow to leave nest

         c) store data to disk (append to hdf)

VISUALIZATION MODULE
1. load configuration file (ini), geometry (csv, numpy) and simulation data (hdf)
2. iterate recorded steps
      - render various images in vertical and horizontal aspect (png)
      - gather statistical information (csv)
3. generate video files (mp4) from image frames (png)
4. generate statistical plots and evaluations
```

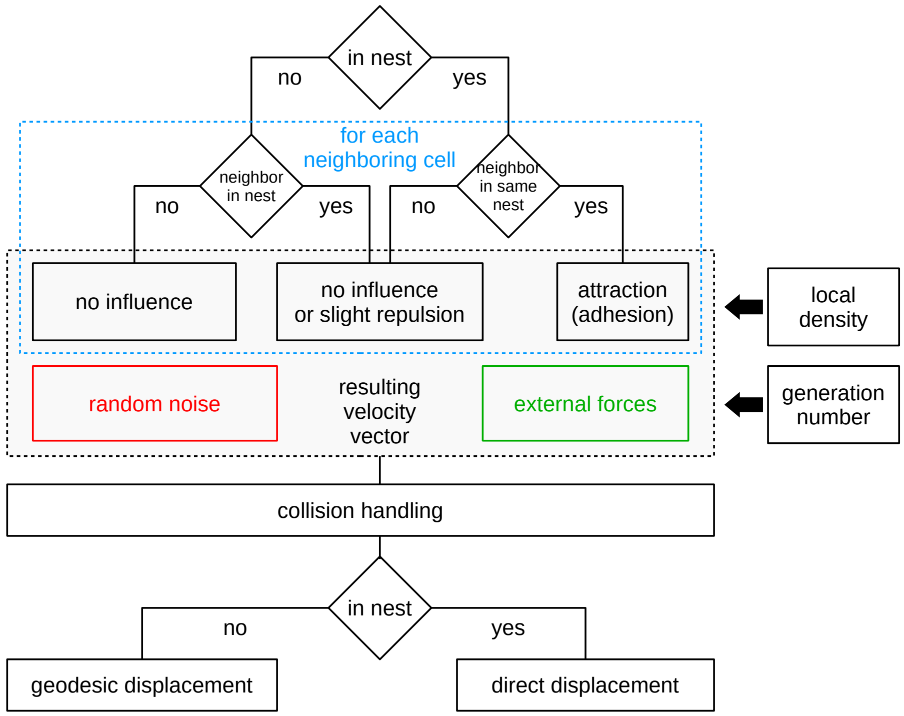

Figure A. Program routine for simulating cell movement. Different intercellular forces apply depending on whether the cell and the neighboring cell are included in a nest. From the intercellular forces and additional random and external forces, a velocity vector is derived. After handling of collisions, the corrected velocity vector is used to displace the cell according to its current configuration. The mathematical model and formulas are presented thoroughly in *Methods* in the main text. A visual outline of the construction of the velocity vector, collision handling and geodesic displacement are provided in Fig 3 in the main text.

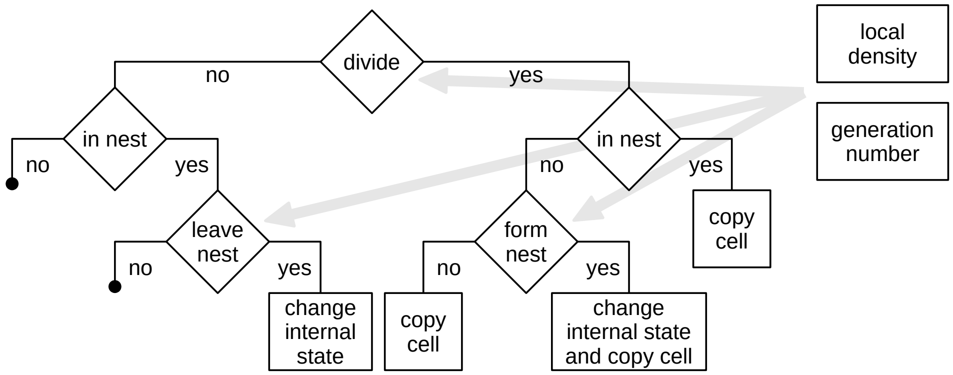

Figure B. Program routine for stochastic events on the cell level. The per step (and conditional) probabilities for a cell to divide, form a nest or leave a nest are modulated by the cell generation number and the local density. Compare the mathematical formula in *Methods* in the main text and also in S3 Text and in S4 Text. Membership in nests or globules is handled internally by a cellular state variable. To implement symmetric cell division, the internal generation number of a cell is increased by one and a hard copy of the cell object with slightly randomized state (position and base probabilities) is generated.

Overview and key elements of the implementation

The simulator and the auxiliary framework were implemented in the Python programming language. For parameterization we use configuration files and a rudimentary scripting language for the definition of control functions. To increase the computational performance of simulations, parallelization and spatial indexing techniques were applied and some critical functions were offloaded as C++ libraries (Table A). The latter concerns especially the geometric transformations and geodesic displacements that are required for simulating the interaction between melanocytes and the basement membrane (Listing B and Listing C).

Table A. Code statistics generated with the tool *cloc*. The numbers are the respective lines of code.

Language	Files	Blank	Comment	Code
Python	55	3897	1117	7104
INI	159	3885	0	7027
C++	2	457	170	1729
Bourne Shell	13	107	38	242
R	5	85	21	131
Bourne Again Shell	4	8	3	29
Markdown	1	18	0	23
Dockerfile	1	7	2	15
Sum	240	8464	1351	16300

Listing B. Implementation of the displacement routine for a single cell-agent in Python. In steps 1, 2 and 3 velocity vectors are calculated and the collisions with neighboring melanocytes are handled, in step 4 movement along the basement membrane surface is offloaded to a C++ implementation (see Listing C).

```
def _step_move_default(self, melanocyte, pointer):

    local_geometry = self.geometry.get_shapes(*melanocyte.get_position_scalars_2d())

    melanocyte_position = melanocyte.get_position()
    melanocyte_radius = melanocyte.radius

    # 1) get neighbors and calculate vectors

    pointers = self.get_neighbours(melanocyte, pointer)

    ...

    N = len(pointers)

    neighbours = [self.melanocytes[neigh_pointer] for neigh_pointer in pointers]
    vectors = numpy.zeros((N, 3))
    for i in range(N):
        vectors[i] = neighbours[i].get_position_scalars() - melanocyte_position
    norms = numpy.linalg.norm(vectors, axis=1)

    nonzeros = numpy.nonzero(norms)[0]
    norms = norms[nonzeros]
    vectors = vectors[nonzeros]
    neighbours = [neighbours[n] for n in nonzeros]

    N = len(neighbours)

    if N > 0:
        vectors /= norms.reshape((-1, 1))
```

```
# 2) movement predictor

distances = [None] * N
neighbor_stati = [None] * N
density = 0.0
velocity = numpy.zeros(3)  # NOTE the velocity vector is in units [um/day]

for i in range(N):

    neighbor = neighbours[i]
    distance = norms[i] - neighbor.radius - melanocyte_radius
    distances[i] = distance
    neighbor_status = self.logic.neighbor_status(melanocyte, neighbor)
    neighbor_stati[i] = neighbor_status

    density += self.logic.density_weight(melanocyte, neighbor, distance, neighbor_status)

    force = self.logic.force(melanocyte, neighbor, distance, neighbor_status)
    velocity += vectors[i] * force

density = min(density, 1.0)
melanocyte.density = density

velocity += self.logic.diffusion(melanocyte)
velocity += self.logic.velocity_vertical(
                                melanocyte,
                                local_geometry.get_altitude(*melanocyte_position))

# store (raw/instantaneous) melanocyte velocity before correction and projection
melanocyte.velocity = self.__norm(velocity)  # ?

# 3) movement corrector

for neighbor, vector, distance, neighbor_status in
            zip(neighbours, vectors, distances, neighbor_stati):

    # projection of current velocity vector onto neighbor direction
    # predictor needs multiplication with delta_t

    projection = (velocity[0] * vector[0] + velocity[1] * vector[1] + velocity[2] * vector[2]) \
                 * self.logic.time_delta

    # overlapping but relaxing
    if distance < projection < 0.0:

        # velocity increase in order to resolve overlap
        # correction factor sign [ sgn(projecteion - distance) ] is negative
        velocity -= vector * (projection - distance) \
            * 0.5 * self.logic.correction_factor(melanocyte, neighbor, density, neighbor_status) \
            / self.logic.time_delta

    # overlapping and colliding
    elif distance < 0.0 <= projection:

        # in order to resolve overlap
        # revert the sign of the velocity component
        velocity -= vector * (projection - distance) \
            * 0.5 * self.logic.correction_factor(melanocyte, neighbor, density, neighbor_status) \
            / self.logic.time_delta

    # nonoverlapping but colliding
    elif 0.0 <= distance < projection:

        # just reduce projected velocity component
        # never increases total velocity
```

```
            # correct by (projection - distance)
            velocity -= vector * (projection - distance) \
                * 0.5 * self.logic.correction_factor(melanocyte, neighbor, density, neighbor_status) \
                / self.logic.time_delta

    # scale velocity
    velocity *= self.logic.time_delta

    # 4) pick from different movement implementations

    if melanocyte.state == 0:
        new_position, _ = local_geometry.move_on_surface(
            *melanocyte.get_position_scalars(), velocity,
            mode=self.config.simulator_surface_movement)
    elif melanocyte.state == 1:
        new_position = local_geometry.move_freely(*melanocyte.get_position_scalars(), velocity)
    elif melanocyte.state == 2:
        new_position = local_geometry.move_freely(*melanocyte.get_position_scalars(), velocity)
    else:
        raise ValueError("unknown melanocyte state value")

    melanocyte.set_position(new_position)
```

Listing C. Excerpt of the implementation of the Papilla class in C++. The code shows the initialization of the ODE solver, which is used to solve the geodesic differential equation (functions `rhs` and `jac`). The functions `translate_parametric_cartesian` and `translate_geodesic` calculate the displacement of cell-agents along the Papilla surface (geometry) using the ODE solver.

```
#include <boost/python.hpp>
#include <gsl/gsl_vector.h>
#include <gsl/gsl_matrix.h>
#include <gsl/gsl_odeiv2.h>

// Papilla class ...

typedef struct
{
    Papilla* papilla;
    PapillaLocalGeometryPolar* local_geometry;
    double A;
    double B;
    double C;
} ode_params_t;

ode_params_t ode_params;
gsl_odeiv2_system ode_system = {rhs, jac, 4, &ode_params};
gsl_odeiv2_step_type* ode_stepper;
gsl_odeiv2_driver* ode_driver;

static inline void update_ode_params(ode_params_t* ode_params,
   const double alpha, const double theta)
{
    PapillaLocalGeometryPolar* local_geometry = ode_params->local_geometry;

    // these checks must not return in the first step!
    if ((local_geometry->alpha == alpha) and (local_geometry->theta == theta)) return;

    ode_params->papilla->update_local_geometry_alpha(*local_geometry, alpha);
    ode_params->papilla->update_local_geometry_theta(*local_geometry, theta);

    const double r = local_geometry->r;
    const double dr = local_geometry->dr;
    const double ddr = local_geometry->ddr;
    // const double h = local_geometry->h;
```

```
    const double dh = local_geometry->dh;
    const double ddh = local_geometry->ddh;

    const double N = 1.0 / (dr * dr + dh * dh);

    ode_params->A = 2.0 * dr / r;
    ode_params->B = dr * r * N;
    ode_params->C = (dr * ddr + dh * ddh) * N;
}

static int rhs(double t, const double y[], double dydt[], void *params)
{
    // abort solver when papilla top is approached
    if (y[2] < THETA_ORIGIN)
        return GSL_EBADFUNC;

    ode_params_t* ode_params = (ode_params_t*) params;
    update_ode_params(ode_params, y[0], y[2]);

    dydt[0] = y[1];
    dydt[1] = - ode_params->A * y[1] * y[3];
    dydt[2] = y[3];
    dydt[3] = ode_params->B * y[1] * y[1] - ode_params->C * y[3] * y[3];

    return GSL_SUCCESS;
}

static int jac(double t, const double y[], double *dfdy, double dfdt[], void *params)
{
    ode_params_t* ode_params = (ode_params_t*) params;
    update_ode_params(ode_params, y[0], y[2]);

    gsl_matrix_view dfdy_mat = gsl_matrix_view_array(dfdy, 4, 4);
    gsl_matrix* jacobian = &dfdy_mat.matrix;

    gsl_matrix_set_zero(jacobian);  // not required?
    gsl_matrix_set(jacobian, 0, 1, 1.0);
    gsl_matrix_set(jacobian, 1, 1, - ode_params->A * y[3]);
    gsl_matrix_set(jacobian, 1, 3, - ode_params->A * y[1]);
    gsl_matrix_set(jacobian, 2, 3, 1.0);
    gsl_matrix_set(jacobian, 3, 1, 2.0 * ode_params->B * y[1] - 2.0 * ode_params->C * y[3]);
    gsl_matrix_set(jacobian, 3, 3, 1.0);

    return GSL_SUCCESS;
}

// Papilla class constructor ...

gsl_odeiv2_step_type* ode_stepper = (gsl_odeiv2_step_type*) gsl_odeiv2_step_rk2;
ode_params.papilla = &(*this);
ode_driver = gsl_odeiv2_driver_alloc_y_new(&ode_system, ode_stepper,
    1e-2 /* initialstep */, 1e-1 /* abserr */, 1e-3 /* relerr */);
gsl_odeiv2_driver_set_hmin(ode_driver, 1e-3 /* minstep */);
gsl_odeiv2_driver_set_hmax(ode_driver, 0.2 /* maxstep */);

// Papilla class parametric translation in Cartesian coordinates ...

boost::python::tuple translate_parametric_cartesian(
        PapillaLocalGeometryCartesian local_geometry,
        const double vx,
        const double vy,
        const double vz,
        const double delta_t)
{
    double x_bar = local_geometry.theta * local_geometry.cos;
    double y_bar = local_geometry.theta * local_geometry.sin;
```

```
    const boost::python::tuple velocity_pspace = local_geometry._pushforward(vx, vy, vz);
    const double vx_bar = boost::python::extract<double>(velocity_pspace[0]);
    const double vy_bar = boost::python::extract<double>(velocity_pspace[1]);

    x_bar += delta_t * vx_bar;
    y_bar += delta_t * vy_bar;

    const double theta = std::sqrt(x_bar * x_bar + y_bar * y_bar);
    update_local_geometry_alpha(local_geometry, x_bar / theta, y_bar / theta);
    update_local_geometry_theta(local_geometry, theta);

    const boost::python::tuple velocity = boost::python::make_tuple(vx, vy, vz);
    return boost::python::make_tuple(
            local_geometry,
            velocity
        );
}

// Papilla class geodesic translation in polar coordinates ...

boost::python::tuple translate_geodesic(
        PapillaLocalGeometryPolar local_geometry,
        const double vx,
        const double vy,
        const double vz,
        const double delta_t)
{
    const double alpha = local_geometry.alpha;
    const double theta = local_geometry.theta;

    const boost::python::tuple velocity_pspace = local_geometry._pushforward(vx, vy, vz);
    const double valpha = boost::python::extract<double>(velocity_pspace[0]);
    const double vtheta = boost::python::extract<double>(velocity_pspace[1]);

    double t = 0.0;
    double y[4] = {alpha, valpha, theta, vtheta};

    // change alpha and theta values so that local_geometry will be updated in the first step
    local_geometry.alpha = - 1.0;
    local_geometry.theta = - 1.0;

    ode_params.local_geometry = &local_geometry;
    const int err = gsl_odeiv2_driver_apply(ode_driver, &t, delta_t, y);
    gsl_odeiv2_driver_reset(ode_driver);

    // solver aborts if papilla top is approached!
    // the origin is a saddle point that cannot be treated in polar coordinates
    // hence, use Cartesian transform (is flat area anyways)
    if ( err == GSL_EBADFUNC )
    {
        PapillaLocalGeometryCartesian local_geometry_2;
        update_local_geometry_alpha(local_geometry_2, alpha);
        update_local_geometry_theta(local_geometry_2, theta);

        return translate_parametric_cartesian(local_geometry_2, vx, vy, vz, delta_t);
    }

    if ( err != GSL_SUCCESS )
    {
        PyErr_SetString(PyExc_RuntimeError, "Integrator failed!");
    }

    update_local_geometry_alpha(local_geometry, y[0]);  // alpha
    update_local_geometry_theta(local_geometry, y[2]);  // theta
```

```
    const boost::python::tuple velocity = local_geometry._pullback(y[1], y[3]);  // (valpha, vtheta)

    return boost::python::make_tuple(
           local_geometry,
           velocity
       );
}
```

In the current optimization stage, a typical simulation run (time span of 1,500 to 2,000 days) of a medium size lesion ($< 10^6$ melanocytes) takes approximately one day on a modern computer system. For reproducibility and subsequent visualization, all information produced by the simulator is stored in HDF5 data files, taking up to 10 GB of disk space for each simulation run. Visualization and animation is implemented with basic 2D rendering techniques in the Python programming language.
